# KuJiang GanLuoYin Alleviates Hypertensive Vascular Injury and Modulates FMO2/FTO/m^6^A Signaling

**DOI:** 10.3390/biomedicines14071469

**Published:** 2026-06-28

**Authors:** Tong Sun, Jianghong Li, Ruijie Shi, Haitao Xie, Siyuan Yin, Xueqian Liu, Shi Wang, Jiandong Chen, Shuhua Tang, Xiaohu Chen

**Affiliations:** 1 The First Clinical School of Medicine, Nanjing University of Chinese Medicine, 138 Xianlin Road, Nanjing 210023, China; suntong246@163.com (T.S.);; 2 Department of Cardiology, Nanjing University of Chinese Medicine, 155 Hanzhong Road, Nanjing 210029, China

**Keywords:** hypertension, FMO2, m^6^A methylation, vascular remodeling, KuJiang GanLuoYin, linarin

## Abstract

**Background**: Hypertension-induced vascular injury involves endothelial dysfunction, inflammation, and oxidative stress, leading to vascular remodeling and cardiovascular complications. Flavin-containing monooxygenase 2 (FMO2) has been implicated in redox regulation, but its role in hypertensive vascular injury remains unclear. This study investigated whether KuJiang GanLuoYin (KJGLY) protects against hypertensive vascular injury and whether FMO2-associated Fat mass and obesity-associated protein (FTO)/N6-methyladenosine (m^6^A) signaling is involved. **Methods**: Spontaneously hypertensive rats (SHRs) were treated with KJGLY for eight weeks. Blood pressure, vascular remodeling, inflammation, oxidative stress, and global m^6^A RNA methylation were assessed. Integrated metabolomic and proteomic analyses were performed to identify treatment-associated molecular alterations and candidate proteins. AAV9-mediated FMO2 knockdown in SHRs and gain- and loss-of-function approaches in angiotensin II (Ang II)-stimulated human umbilical vein endothelial cells were used to examine the functional involvement of FMO2. Ultra-performance liquid chromatography coupled with quadrupole time-of-flight mass spectrometry (UPLC-Q-TOF/MS)-based chemical profiling and High-performance liquid chromatography–tandem mass spectrometry (HPLC–MS/MS) quantification were performed to characterize the major constituents of KJGLY. **Results**: KJGLY significantly reduced blood pressure and alleviated vascular remodeling in SHRs. Metabolomic and proteomic analyses revealed treatment-associated alterations in inflammatory and lipid metabolic pathways and identified FMO2 as a treatment-responsive candidate. KJGLY restored FMO2 expression, reduced FTO abundance and NF-κB activation, increased global m^6^A levels, and attenuated inflammatory and oxidative stress responses in hypertensive aortas. Conversely, AAV9-mediated FMO2 knockdown aggravated vascular injury, enhanced inflammation and oxidative stress, reduced global m^6^A levels, and increased NF-κB activation. Co-immunoprecipitation showed an association between FMO2 and FTO, and MeRIP-qPCR indicated that FMO2 manipulation altered m^6^A enrichment of VCAM-1 mRNA. In Ang II-stimulated endothelial cells, linarin, the most abundant quantified constituent of KJGLY, partially recapitulated the cellular effects of KJGLY, including restoration of FMO2/FTO-associated signaling and attenuation of inflammatory activation. **Conclusions**: These findings support a functional role for FMO2 in hypertensive vascular injury and suggest that FMO2-associated modulation of FTO/m^6^A signaling may contribute to the vascular protective effects of KJGLY. Linarin recapitulated key protective effects in vitro, although its in vivo contribution to the formula remains to be determined.

## 1. Introduction

Hypertension is one of the leading modifiable risk factors for cardiovascular disease and mortality worldwide [1]. Despite the availability of effective antihypertensive therapies, the global burden of uncontrolled hypertension remains substantial [2]. Persistent elevation of blood pressure is associated with endothelial dysfunction and structural remodeling of both resistance and conduit arteries, thereby contributing to increased vascular stiffness and cardiovascular complications. Oxidative stress and inflammation are central to these pathological changes and can further impair vascular function, establishing a self-reinforcing cycle of vascular injury, remodeling, and sustained hypertension [3,4]. Increasing evidence therefore indicates that vascular injury is not only a consequence of hypertension but also an important driver of disease progression, highlighting the need to identify molecular pathways involved in the maintenance of vascular homeostasis during hypertension.

N6-methyladenosine (m^6^A) is the most abundant internal modification of eukaryotic messenger RNA and regulates multiple aspects of RNA metabolism, including splicing, stability, translation, and degradation [5]. The dynamic balance of m^6^A is controlled by methyltransferases, demethylases, and m^6^A-binding proteins. Fat mass and obesity-associated protein (FTO) is one of the best-characterized m^6^A demethylases and has been implicated in multiple cardiovascular and metabolic disorders [6]. Kruger et al. demonstrated that endothelial FTO deficiency protected against obesity-induced hypertension and preserved vascular integrity [7]. Although FTO has been implicated in vascular dysfunction, the upstream factors associated with FTO-dependent m^6^A signaling during hypertensive vascular injury remain poorly understood. Traditional Chinese Medicine (TCM) is widely used as a complementary therapeutic approach for hypertension and hypertension-related organ injury, and several herbal formulas have shown potential vascular protective effects [8,9]. KuJiang GanLuoYin (KJGLY) is a six-herb formula comprising Kuding Tea (Folium Ilicis Kudingcha, KDC), Wild Chrysanthemum (Flos Chrysanthemi Indici, YJH), Hairy Beggarticks (Herba Bidentis Pilosae, GZC), Chuanxiong Rhizome (Rhizoma Chuanxiong, CX), Kudzu Root (Radix Puerariae, GG), and Stevia Leaf (Folium Steviae, TYJ). Previous studies have reported that these medicinal herbs and their constituents possess antihypertensive, anti-inflammatory, antioxidant, and endothelial protective activities [10,11,12,13,14,15]. However, the pharmacologically relevant constituents and molecular pathways associated with the vascular effects of KJGLY remain incompletely characterized.

In the present study, we evaluated the effects of KJGLY on hypertension-associated vascular injury in spontaneously hypertensive rats (SHRs). Integrated metabolomic and proteomic analyses were used to characterize treatment-associated molecular alterations and screen for candidate proteins. Based on the proteomic findings, FMO2 was selected for subsequent in vivo and in vitro functional investigation. Particular attention was given to the role of FMO2-associated m^6^A signaling in the regulation of vascular inflammation and oxidative stress. In addition, linarin, the most abundant quantified constituent of KJGLY, was selected as a representative candidate compound for exploratory validation in endothelial cells.

## 2. Materials and Methods

### 2.1. KJGLY Preparation and UPLC-Q-TOF/MS Analysis

All herbal ingredients were provided by the Pharmaceutical Preparation Laboratory of Traditional Chinese Medicine at the Jiangsu Provincial Hospital of Traditional Chinese Medicine and were authenticated by two senior herbalists. The components were weighed in a 30-30-30-15-15-8 ratio. The detailed steps are described in the Supplementary Materials (Supplementary Methods Section).

### 2.2. Quantitative HPLC-MS/MS Analysis of Representative Constituents in KJGLY

An aliquot of the same concentrated KJGLY aqueous decoction prepared as described in Section 2.1 was used for quantitative HPLC-MS/MS analysis. Sample pretreatment was performed using the same ethanol precipitation, vacuum concentration, and reconstitution procedures described above (Supplementary Methods Section).

### 2.3. Animals

SHRs and age-matched Wistar–Kyoto (WKY) rats were housed under standard laboratory conditions with free access to food and water. SHRs were randomly assigned to receive vehicle, valsartan, or KJGLY at low and high doses for eight weeks, while WKY rats served as normotensive controls. Systolic blood pressure was monitored throughout the study. At the end of the treatment period, blood and aortic tissues were collected for subsequent biochemical, histological, and molecular analyses. Detailed experimental procedures are provided in the Supplementary Methods.

### 2.4. Label-Free Quantitative Proteomics

Proteomic analysis was performed using rat aortic tissues. Total proteins were extracted and quantified prior to enzymatic digestion. The resulting peptides were purified and subjected to nano-liquid chromatography coupled with quadrupole-Orbitrap tandem mass spectrometry using a Q Exactive mass spectrometer (Thermo Scientific, Waltham, MA, USA) coupled to a nano-C18 separation column. Peptide spectra were acquired in data-dependent acquisition mode and searched against the UniProt Rattus norvegicus database using MaxQuant software (v1.6.14). Protein identification was performed with a false discovery rate (FDR) threshold of 1%, and label-free quantification (LFQ) values were used for downstream analyses.

### 2.5. High-Resolution Non-Targeted Metabolomics

Untargeted metabolomic analysis of aortic tissues was performed using UHPLC-MS. Metabolites were extracted with methanol/acetonitrile/water, followed by protein precipitation, centrifugation, and sample reconstitution. Chromatographic separation was achieved using an Agilent 1290 UHPLC system (Agilent Technologies, Santa Clara, CA, USA) coupled to an AB Triple TOF 6600 mass spectrometer (SCIEX, Framingham, MA, USA) operating in both positive and negative ionization modes. Quality control samples were included throughout the analytical run to ensure data stability.

### 2.6. Eukaryotic Reference Transcriptomics

Transcriptomic profiling was performed using aortic tissues. Total RNA was isolated and subjected to quality assessment prior to library construction and paired-end sequencing on an Illumina NovaSeq 6000 platform (San Diego, CA, USA). Following stringent quality control, the resulting clean reads were aligned with the reference genome. A functional annotation and pathway enrichment analysis were then carried out to pinpoint the biological processes associated with AAV9-mediated FMO2 knockdown.

### 2.7. Cell Culture and Plasmid Transfection

Human umbilical vein endothelial cells (HUVECs; RRID: CVCL_2959) were cultured under standard conditions. FMO2 and FTO gain- and loss-of-function experiments were performed using plasmids or siRNA obtained from OBIO (Shanghai, China). Detailed experimental procedures are described in the Supplementary Methods.

### 2.8. Measurement of m^6^A Content and Methylated RNA Immunoprecipitation with qPCR (MeRIP-qPCR)

Global m^6^A levels in RNA were measured using the EpiQuik™ m^6^A RNA methylation quantification kit (Epigentek, Farmingdale, NY, USA). To assess m^6^A modification on *VCAM-1* transcripts, m^6^A-RIP was performed. Briefly, m^6^A antibodies and control IgG were conjugated to protein A/G magnetic beads (RiboBio, Guangzhou, China) at 4 °C overnight. Total RNA (Takara Bio Inc., Shiga, Japan) was extracted, fragmented, and incubated with beads in RNase inhibitor-containing IP buffer for 3 h at 4 °C. After two washes, the immunoprecipitated RNA was eluted, purified, and subjected to reverse transcription. Relative expression in input and IP fractions was quantified by RT-qPCR (Takara Bio Inc., Shiga, Japan).

### 2.9. Quantitative Real-Time PCR (RT-qPCR)

Total RNA was extracted, reverse-transcribed into cDNA, and amplified using a SYBR Green detection system. Relative transcript abundance was normalized to GAPDH and calculated using the 2^−ΔΔCt^ method. Primer sequences are listed in Table S3.

### 2.10. Western Blot Analysis and Co-Immunoprecipitation

Total proteins were isolated from tissues or cells using RIPA lysis buffer. Protein bands were visualized using enhanced chemiluminescence reagents. β-Actin or GAPDH was used as the internal loading control.

For co-immunoprecipitation analysis, HUVEC lysates were incubated with an anti-FMO2 antibody at 4 °C overnight, followed by capture with Protein A/G agarose beads. Immunoprecipitated proteins were subsequently analyzed by Western blotting.

### 2.11. Cell Viability Assay

Cell viability was determined using the CCK-8 assay according to the manufacturer’s instructions.

### 2.12. Enzyme-Linked Immunosorbent Assay

Serum IL-1β, IL-6, and IL-10 concentrations were measured using rat ELISA kits according to the manufacturer’s instructions (Elabscience, Houston, TX, USA).

### 2.13. Assays for Malondialdehyde, Glutathione, Superoxide Dismutase, and Nitric Oxide Levels

Oxidative stress status in aortic tissues was evaluated by measuring malondialdehyde (MDA), glutathione (GSH), superoxide dismutase (SOD), and nitric oxide (NO) levels using commercially available assay kits (Beyotime, Shanghai, China). Measurements were performed following the manufacturers’ recommended protocols.

### 2.14. Histopathological Analyses

Aortic tissues were fixed, paraffin-embedded, and processed for histological and immunostaining analyses. H&E and Masson’s trichrome staining were performed to evaluate vascular morphology and collagen deposition, respectively. Vascular wall thickness and fibrotic areas were quantified using image analysis software.

Immunohistochemistry was conducted to assess the expression of VCAM-1 and ICAM-1, whereas immunofluorescence staining was performed to examine FMO2 and FTO expression in aortic tissues. Detailed staining procedures and antibody information are provided in the Supplementary Methods.

### 2.15. Statistical Analysis

All data are expressed as mean ± SD. Statistical analyses were conducted using GraphPad Prism 9.0. Comparisons between two groups were performed using Student’s *t*-test, while multiple-group comparisons were analyzed by one-way ANOVA. Statistical significance was defined as *p* < 0.05.

## 3. Results

### 3.1. Chemical Characterization of KJGLY by UPLC-Q-TOF/MS

KJGLY comprises six natural medicinal ingredients (Table 1). The chemical composition of KJGLY was characterized using UPLC-Q-TOF/MS. Representative base peak chromatograms acquired in positive and negative ion modes are shown in Figure S1A,B. Several retention time-aligned peaks (Figure S1C,D), such as peaks 14 and 43 were consistently observed in both sample and standard chromatograms, suggesting the presence of these compounds in KJGLY. In total, 73 chemical constituents were characterized in KJGLY, including 27 flavonoid components, 13 organic acid components, 8 saponin components, 6 phthalide components, 4 isoflavone components, 2 coumarin components, and 13 additional miscellaneous components (Table S1). To assist in identification, a mixed standard solution containing gallic acid, chlorogenic acid, ferulic acid, and quercetin was analyzed under the same conditions (Table S2). These findings provide the chemical basis for the subsequent pharmacological investigation of KJGLY.

### 3.2. KJGLY Reduces Blood Pressure and Attenuates Vascular Remodeling in SHRs

**Figure 1:**
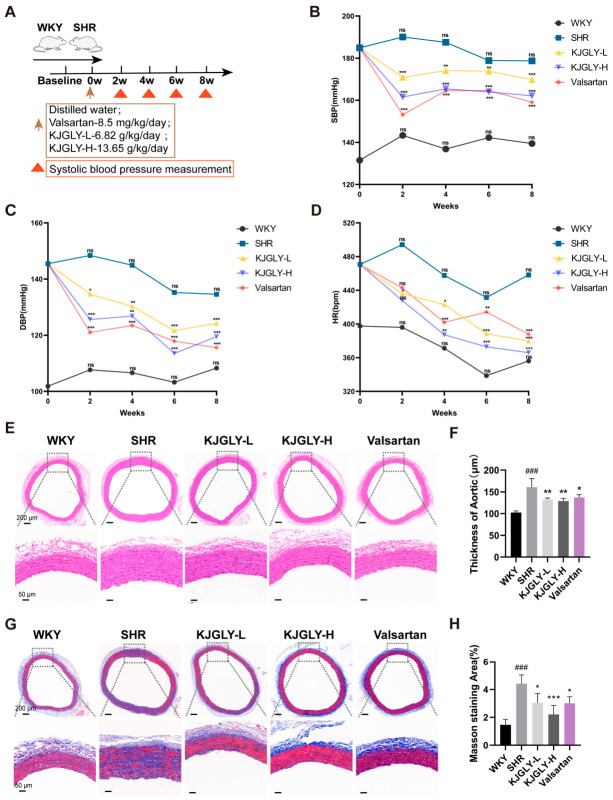
The Effects of KJGLY Treatment on Blood Pressure, Aortic Remodeling, and Endothelial Integrity in SHRs. (**A**) Experimental timeline and treatment groups: SHRs received KJGLY at low or high doses, or valsartan, via oral gavage for 8 weeks. (**B**–**D**) SBP, DBP, and HR were measured at baseline and every two weeks thereafter. (**E**) Representative H&E staining images of rat aorta. Scale bar: 200 μm, 50 μm. (**F**) Aortic wall thickness was measured by H&E staining. (**G**) Representative Masson’s staining images of rat aorta. Scale bar: 200 μm, 50 μm. (**H**) Masson staining was used to assess collagen deposition in the aorta. All data are presented as mean ± SD (*n* = 6); * *p* < 0.05, ** *p* < 0.01, *** *p* < 0.001 vs. 0 week within the same group; * *p* < 0.05, ** *p* < 0.01, *** *p* < 0.001 vs. SHR group; ### *p* < 0.001 vs. WKY group; ns, no significant.

The experimental design for evaluating the antihypertensive effects of KJGLY in SHRs is shown in Figure 1A. Compared with untreated SHRs, KJGLY treatment significantly reduced systolic blood pressure (SBP) and diastolic blood pressure (DBP) at 2, 4, 6, and 8 weeks; the high-dose KJGLY group (KJGLY-H) showed consistently lower SBP and DBP values than untreated SHRs throughout the intervention period (Figure 1B,C). A significant reduction in blood pressure was observed in the valsartan-treated group, suggesting that KJGLY exhibits antihypertensive efficacy analogous to that of this first-line clinical agent. The heart rate (HR) was lower in the KJGLY-H group than in untreated SHRs during the treatment period (Figure 1D). Histological analysis (Figure 1E,F) revealed marked aortic wall thickening and inflammatory cell infiltration in untreated SHRs compared with WKY rats. In contrast, both KJGLY-L and KJGLY-H treatments attenuated aortic wall thickening and improved aortic histological morphology. Similar histological improvements were observed in the valsartan-treated group, supporting the vascular protective effects of KJGLY. Masson’s trichrome staining (Figure 1G,H) further showed increased collagen deposition in the aortic wall of untreated SHRs, whereas KJGLY treatment significantly reduced collagen accumulation, with a more evident reduction in the high-dose group. Collectively, these results indicate that KJGLY lowers blood pressure and attenuates vascular wall thickening and collagen deposition in SHRs.

### 3.3. KJGLY Modulates Inflammation-Associated Metabolic Profiles in Hypertensive Aorta

**Figure 2:**
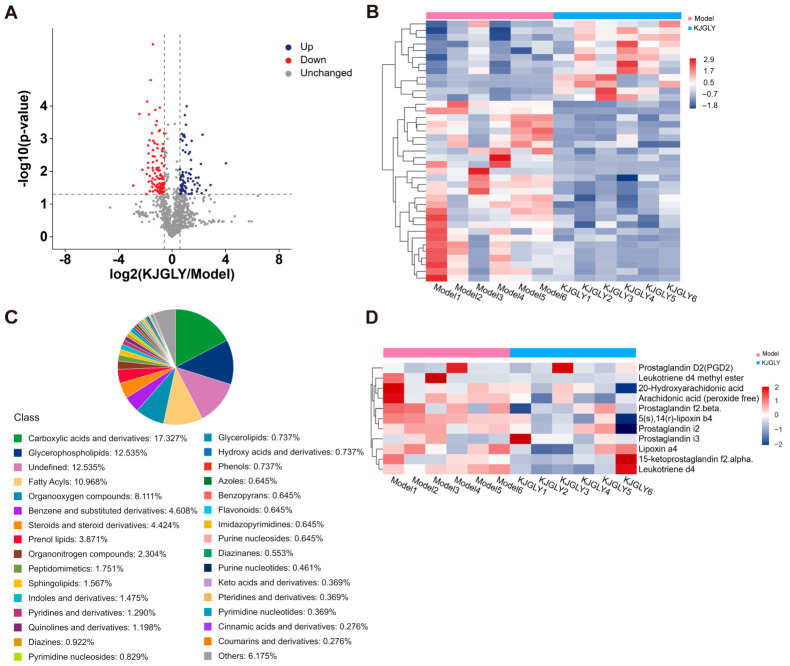
Integrated metabolomics analysis of KJGLY treatment in SHRs. (**A**) Volcano plot showing significantly altered metabolites (red: upregulated, blue: downregulated). (**B**) Heatmap of differential metabolites clustered by sample and metabolite expression levels. (**C**) Pie chart summarizing the chemical classification of the identified metabolites. (**D**) Heatmap of selected inflammation-related metabolites, including prostaglandins, leukotrienes, and lipoxins.

To characterize the metabolic alterations associated with KJGLY treatment, untargeted metabolomic profiling was performed using aortic tissues from untreated SHRs and KJGLY-H-treated SHRs. Because KJGLY-H produced the most pronounced antihypertensive and vascular protective effects, this group was selected for subsequent omics analysis. Volcano plot and hierarchical clustering analyses revealed distinct metabolite profiles between the Model and KJGLY-H groups, with clear separation of the biological samples according to treatment (Figure 2A,B). Chemical classification analysis showed that the differential metabolites were predominantly assigned to lipids and lipid-like molecules, organic acids and their derivatives, and organoheterocyclic compounds (Figure 2C). KEGG pathway enrichment analysis indicated that these metabolites were associated with several metabolic and signaling pathways, including arachidonic acid metabolism, linoleic acid metabolism, retrograde endocannabinoid signaling, and ferroptosis (Figure S2A). Pairwise correlation analysis further revealed extensive positive and negative associations among the differential metabolites, indicating coordinated variation within the metabolomic dataset following KJGLY-H treatment (Figure S2B). Consistent with the enrichment of lipid-related pathways, representative arachidonic acid-derived lipid mediators, including prostaglandins, leukotrienes, lipoxins, and related eicosanoid metabolites, exhibited distinct and bidirectional abundance patterns between the Model and KJGLY-H groups (Figure 2D). These results indicate that KJGLY treatment is associated with alterations in lipid mediator profiles, particularly those related to arachidonic acid metabolism, in hypertensive aortic tissue.

### 3.4. Proteomics and Multi-Omics Link FMO2 to Inflammation and Metabolic Changes in Hypertensive Aorta

**Figure 3:**
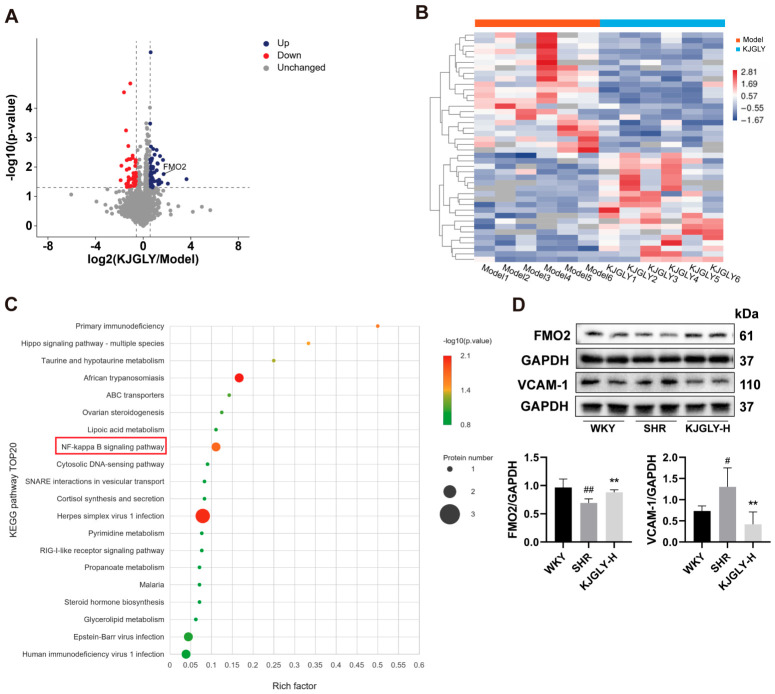
Proteomic Analysis Reveals FMO2 as a Key Candidate Associated with the Vascular Protective Effects of KJGLY. (**A**) Volcano plots of aortic proteomics analysis in the Model and KJGLY-H treatment groups. (**B**) Heatmap representation of protein expression levels in aortic tissue, comparing the Model and KJGLY-H treatment groups. (**C**) Pathway enrichment analysis using KEGG pathways. (**D**) Western blot analysis of FMO2 and VCAM-1 protein expression across different groups. All data are presented as mean ± SD (*n* = 3). # *p* < 0.05, ** *p* < 0.01 vs. WKY group; ## *p* < 0.01 vs. SHR group.

To identify molecular changes associated with the vascular protective effects of KJGLY, we performed quantitative proteomic profiling on aortic tissues from untreated SHRs and KJGLY-treated SHRs. Volcano plot analysis identified a set of differentially expressed proteins between the two groups, and hierarchical clustering separated samples according to treatment, indicating distinct and reproducible proteomic profiles following KJGLY administration (Figure 3A,B). Among these, FMO2 was significantly upregulated by KJGLY treatment and selected for further analysis. KEGG pathway enrichment analysis showed that the differentially expressed proteins were enriched in immune, inflammatory, and metabolic pathways, including NF-κB signaling, arachidonic acid metabolism, lipid metabolism, and ABC transporter-related pathways (Figure 3C). Western blot analysis confirmed the proteomic findings. FMO2 protein expression was lower in SHR aortas than in WKY controls and was significantly restored by KJGLY-H treatment. Conversely, VCAM-1 expression was higher in SHRs and markedly reduced by KJGLY-H administration (Figure 3D). These reciprocal expression patterns suggest an association between FMO2 restoration and reduced vascular inflammation following KJGLY treatment.

GO functional classification assigned the differentially expressed proteins mainly to biological processes related to cellular and metabolic regulation, stimulus response, and immune functions. Major cellular component categories included intracellular and membrane-associated compartments, and the predominant molecular functions were binding and catalytic activity (Figure S3A). To explore the relationship between FMO2 and KJGLY-associated metabolic changes, we constructed an FMO2-centered protein–metabolite association network. FMO2 was associated with multiple differential metabolites, including amino acids and related metabolites (glutamine, glutamic acid, serine, histidine, valine, taurine, argininosuccinic acid, pyroglutamic acid), as well as nicotinamide, PGD2, and LPC (Figure S3B).

Integrated proteomic–metabolomic correlation analysis revealed cross-omics associations between differentially expressed proteins and metabolites. The clustered heatmap showed distinct correlation patterns among selected proteins and metabolites (Figure S4A), and the correlation matrix revealed extensive positive and negative associations involving FMO2, VCAM-1, and metabolites linked to amino acid metabolism, lipid metabolism, and oxidative stress (Figure S4B).

Together, these results identify FMO2 as a treatment-responsive protein associated with inflammatory and metabolic remodeling in the hypertensive aorta.

### 3.5. KJGLY Attenuates Vascular Inflammation and Oxidative Stress in SHRs in Association with FMO2/FTO/m^6^A Signaling

**Figure 4:**
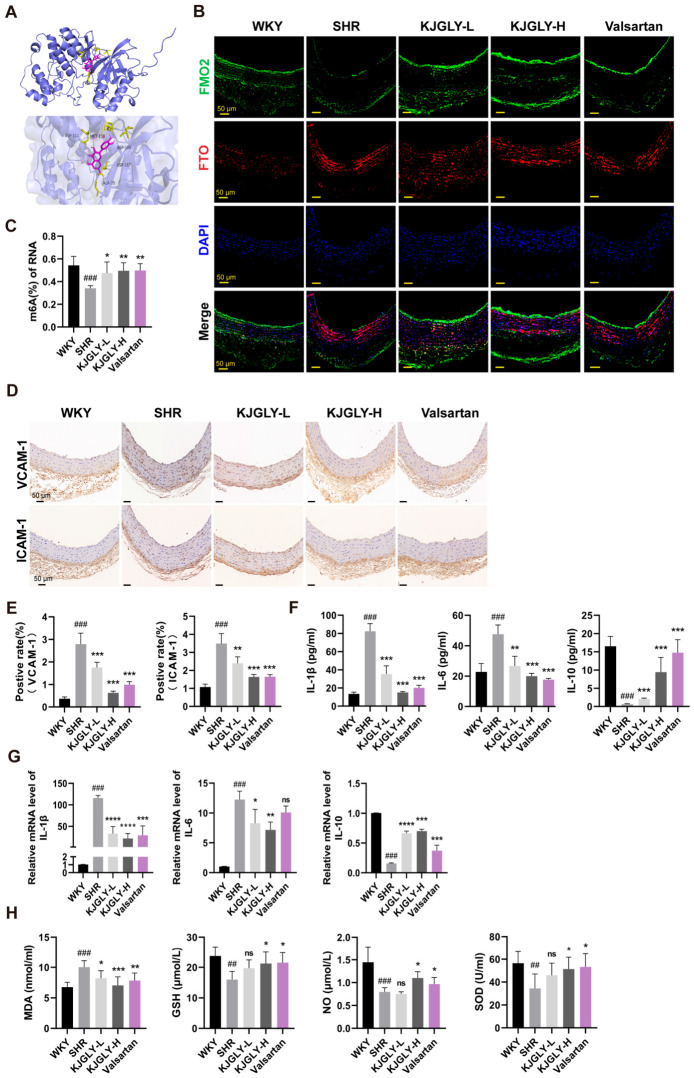
KJGLY Modulates FMO2/FTO-Related m^6^A Signaling and Attenuates Vascular Inflammation in SHRs. (**A**) Molecular docking predicts the binding of FMO2 to FTO. (**B**) Immunofluorescence staining for FMO2 (green), FTO (red), and nuclear staining with DAPI (blue) in the aortic sections. Scale bar: 50 μm. (**C**) Quantitative analysis of m^6^A levels in aortic tissue. (**D**) Immunohistochemical staining of VCAM-1 and ICAM-1 in aortic sections. Scale bar: 50 μm. (**E**) Quantification of VCAM-1 and ICAM-1 expression in the aortic tissue. (**F**) Measurement of inflammatory cytokines from serum: IL-1β, IL-6, and IL-10. (**G**) The relative mRNA expression levels of *IL-6*, *IL-1β*, and *IL-10* in aortic tissue were analyzed by RT-PCR. (**H**) Measurement of oxidative stress marker from aortic tissue: MDA, GSH, NO levels, and SOD. All data are presented as mean ± SD (*n* = 6); * *p* < 0.05, ** *p* < 0.01, *** *p* < 0.001, **** *p* < 0.0001 vs. SHR group; ## *p* < 0.01, ### *p* < 0.001 vs. WKY group; ns, no significant.

Molecular docking analysis predicted a potential interaction interface between FMO2 and FTO. The docking model yielded an estimated binding free energy of −10.3 ± 0.46 kcal/mol and a predicted Ki value of 26.1 nM (Figure 4A), providing an in silico basis for further investigation of their potential association. The expression patterns of FMO2 and FTO were subsequently examined in aortic tissues. Immunofluorescence staining showed lower FMO2 expression and higher FTO expression in SHRs than in WKY rats. These alterations were partially reversed by KJGLY treatment, with more evident changes observed in the high-dose group (Figure 4B). Consistent with these expression patterns, global m^6^A levels were reduced in SHR aortas and increased following KJGLY administration (Figure 4C).

Immunohistochemical analysis further showed that VCAM-1 and ICAM-1 expression was markedly elevated in aortic tissues from SHRs compared with WKY controls. Treatment with either KJGLY or valsartan significantly reduced the positively stained areas of both adhesion molecules (Figure 4D,E), indicating attenuation of vascular inflammatory activation. Serum cytokine measurements showed that IL-1β and IL-6 levels were increased, whereas IL-10 levels were reduced, in SHRs. KJGLY treatment, particularly at the high dose, decreased circulating IL-1β and IL-6 levels and increased IL-10 levels (Figure 4F). RT-qPCR analysis of aortic tissues showed a similar pattern at the transcriptional level. Compared with WKY rats, SHRs exhibited increased *IL-1β* and *IL-6* mRNA expression and reduced *IL-10* mRNA expression. Both doses of KJGLY, as well as valsartan, reduced *IL-1β* and *IL-6* mRNA levels and increased *IL-10* mRNA levels relative to untreated SHRs (Figure 4G). These findings indicate that KJGLY attenuated both local vascular and systemic inflammatory responses in SHRs. Oxidative stress-related indices were also evaluated in aortic tissues. Compared with untreated SHRs, KJGLY-treated rats exhibited reduced MDA levels, increased GSH and NO levels, and enhanced SOD activity (Figure 4H). These changes were consistent with reduced lipid peroxidation and improved antioxidant status following KJGLY treatment.

Collectively, KJGLY attenuated vascular inflammatory and oxidative stress phenotypes in SHRs. These effects were accompanied by restored FMO2 expression, reduced FTO abundance, and increased global m^6^A levels, supporting a potential association between the vascular protective effects of KJGLY and FMO2/FTO-related m^6^A regulation.

### 3.6. AAV9-Mediated FMO2 Knockdown Aggravates Vascular Injury in SHRs

**Figure 5:**
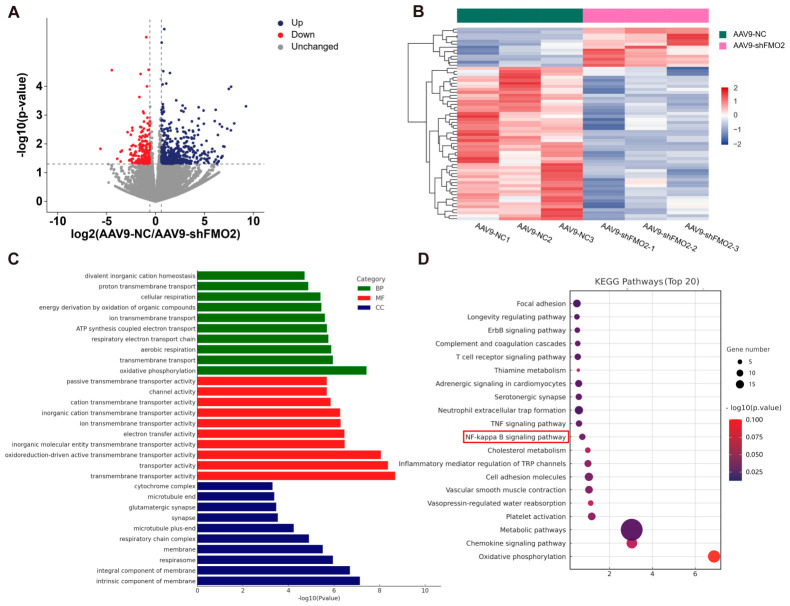
Transcriptomic profiling of aortic tissue following FMO2 knockdown in SHRs. (**A**) Volcano plot showing DEGs between AAV9-shFMO2 and AAV9-NC groups. Upregulated and downregulated genes are indicated in red and blue, respectively. (**B**) Hierarchical clustering heatmap illustrating distinct gene expression patterns between groups. (**C**) GO enrichment analysis of DEGs categorized into BP, CC, and MF terms. (**D**) KEGG pathway enrichment analysis of DEGs.

To further characterize the transcriptional alterations associated with FMO2 knockdown in vivo, RNA sequencing was performed on whole aortic tissues from SHRs systemically administered AAV9 carrying a non-targeting control shRNA (AAV9-NC) or FMO2-targeting shRNA (AAV9-shFMO2). Volcano plot analysis identified multiple differentially expressed genes following FMO2 knockdown (Figure 5A). Hierarchical clustering demonstrated clear separation between the two groups, indicating that AAV9-mediated FMO2 knockdown was associated with broad alterations in the aortic transcriptome (Figure 5B). GO enrichment analysis showed that the differentially expressed genes were predominantly involved in biological processes related to inflammatory response, leukocyte adhesion, cytokine production, and oxidative stress regulation (Figure 5C). KEGG pathway analysis further revealed enrichment of NF-κB signaling, cytokine–cytokine receptor interaction, and cell adhesion molecules (Figure 5D). These results indicate that FMO2 knockdown is associated with transcriptional alterations involving vascular inflammation, immune signaling, and oxidative stress-related processes.

**Figure 6:**
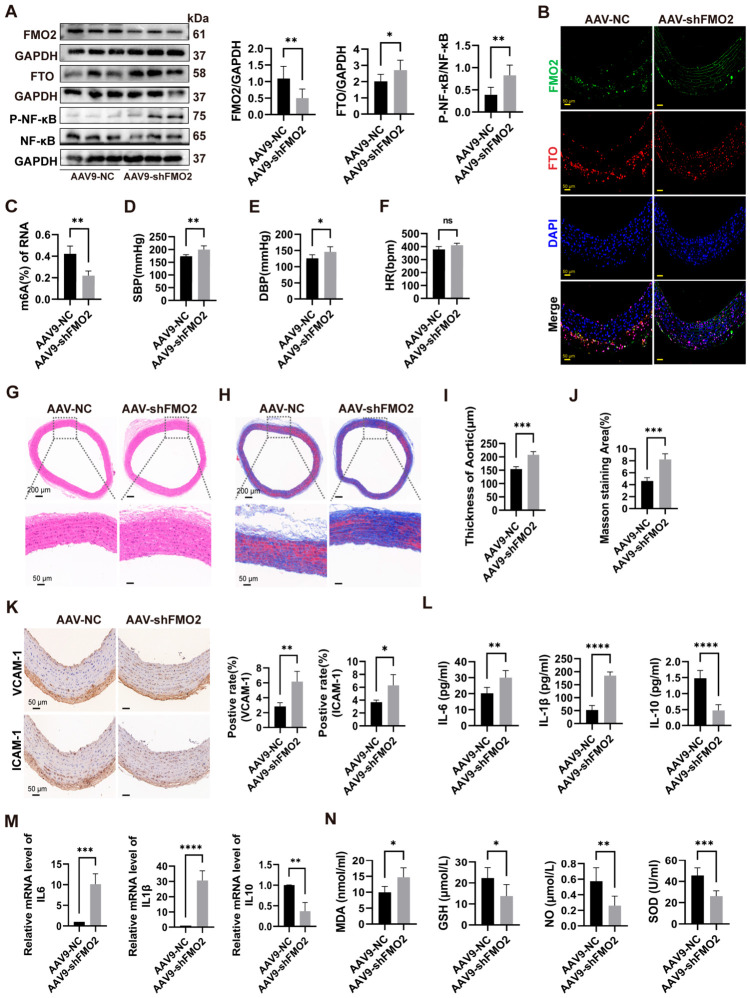
FMO2 Deficiency Promotes Vascular Injury and Inflammation in SHRs. (**A**) Western blot analysis of FMO2, FTO, NF-κB, and p-NF-κB protein expression in aortic tissues from the AAV-NC and AAV-shFMO2 groups (*n* = 4). (**B**) Immunofluorescence staining for FMO2 (green), FTO (red), and nuclear staining with DAPI (blue) in the aortic sections (*n* = 3). (**C**) Quantitative analysis of m^6^A levels in aortic tissues (*n* = 4). (**D**) SBP changes in rats from each group (*n* = 4). (**E**) DBP changes in rats from each group (*n* = 4). (**F**) HR changes in rats from each group (*n* = 4). (**G**) Representative H&E staining of aortic tissues, illustrating structural alterations (*n* = 4). Scale bar: 200 μm, 50 μm. (**H**) Masson’s staining demonstrating collagen deposition in aortic tissues (*n* = 4). Scale bar: 200 μm, 50 μm. (**I**) Quantitative analysis of aortic wall thickness based on H&E staining (*n* = 4). (**J**) Quantification of collagen content from Masson’s trichrome staining (*n* = 4). (**K**) Immunohistochemical staining and quantitative analysis of VCAM-1 and ICAM-1 expression in aortic sections (*n* = 4). (**L**) Levels of inflammatory cytokines (IL-1β, IL-6, IL-10) in serum (*n* = 3). (**M**) The relative mRNA expression levels of *IL-6*, *IL-1β*, and *IL-10* in aortic tissue were analyzed by RT-PCR. (**N**) Quantification of oxidative stress markers (MDA, GSH, NO, and SOD) in aortic tissues (*n* = 4). All data are presented as mean ± SD. * *p* < 0.05, ** *p* < 0.01, *** *p* < 0.001, **** *p* < 0.0001 vs. AAV-NC group; ns, no significant.

The transcriptomic findings were subsequently examined using molecular and phenotypic validation experiments. SHRs were subjected to a single tail vein injection of AAV9-shFMO2 or AAV9-NC. Three weeks after injection, the SHRs were euthanized for experimental analysis. Western blotting confirmed reduced FMO2 expression in the AAV9-shFMO2 group, accompanied by increased FTO expression and NF-κB phosphorylation compared with the AAV9-NC group (Figure 6A). Immunofluorescence staining further showed lower FMO2 and higher FTO signals in aortic tissues of the AAV9-shFMO2 group (Figure 6B). Consistently, global m^6^A abundance was significantly reduced in the AAV9-shFMO2 group (Figure 6C). Hemodynamic measurements showed that FMO2 knockdown significantly increased systolic and diastolic blood pressure, whereas HR remained unchanged (Figure 6D–F). Histological analysis revealed greater aortic wall thickening and increased collagen deposition, as indicated by HE and Masson’s staining (Figure 6G–J). Immunohistochemistry revealed significantly higher VCAM-1 and ICAM-1 expression in the AAV9-shFMO2 group, suggesting enhanced vascular inflammation (Figure 6K). Serum levels of the pro-inflammatory cytokines IL-1β and IL-6 were significantly elevated in rat serum, whereas the anti-inflammatory cytokine *IL-10* was reduced (Figure 6L). RT-qPCR analysis of aortic tissues showed corresponding increases in *IL-1β* and *IL-6* mRNA expression and a reduction in IL-10 mRNA expression (Figure 6M). Assessment of oxidative stress showed increased MDA levels, together with reduced SOD activity and lower GSH and NO levels, in the AAV9-shFMO2 group (Figure 6N), consistent with aggravated lipid peroxidation and impaired antioxidant status. Taken together, these results suggest that suppression of FMO2 exacerbates vascular inflammation, promotes oxidative stress, and worsens vascular injury in SHRs.

### 3.7. Linarin Attenuates Ang II-Induced Endothelial Inflammatory Injury in Association with FMO2/FTO/m^6^A Signaling

**Figure 7:**
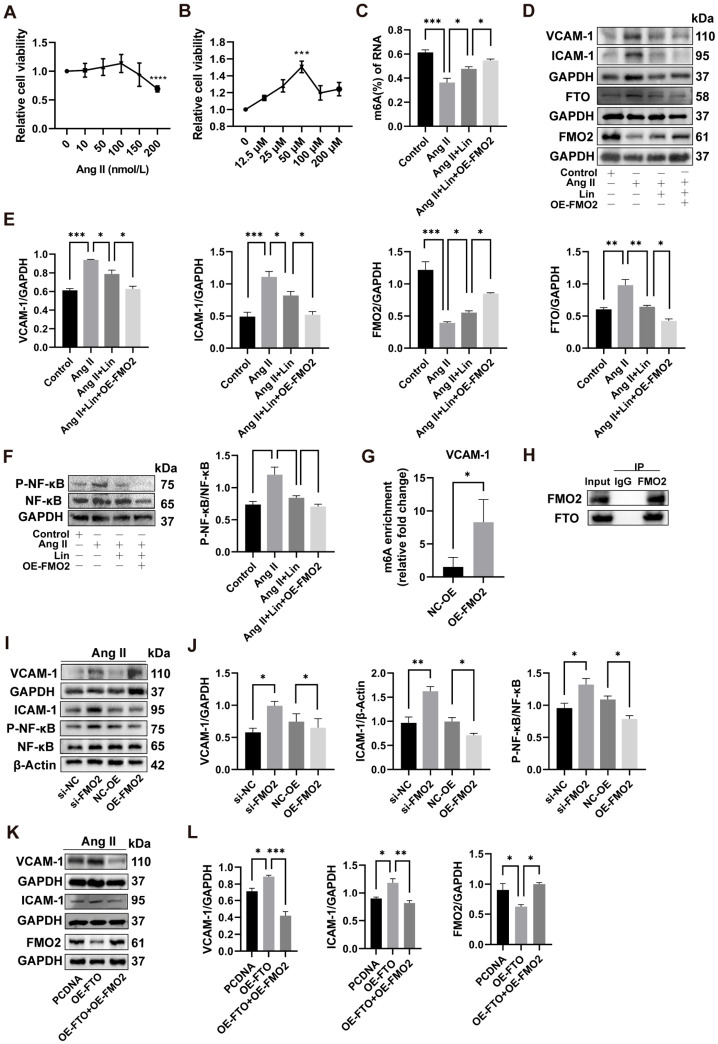
Linarin attenuates Ang II-induced endothelial inflammatory injury in association with FMO2/FTO/m^6^A signaling. (**A**) Effects of different concentrations of Ang II on HUVEC viability determined by CCK-8 assay. (**B**) Effects of linarin (Lin) on the viability of Ang II-injured HUVECs. (**C**) Global m^6^A levels in HUVECs following Ang II stimulation, linarin treatment, and FMO2 overexpression. (**D**) Representative Western blot images showing the expression of VCAM-1, ICAM-1, FTO, and FMO2 in different treatment groups. (**E**) Quantitative analysis of VCAM-1, ICAM-1, FMO2, and FTO protein expression. (**F**) Representative Western blot images and quantitative analysis of NF-κB activation. (**G**) MeRIP-qPCR analysis showing m^6^A enrichment of *VCAM-1* mRNA following FMO2 overexpression. (**H**) Co-immunoprecipitation analysis showing an association between endogenous FMO2 and FTO proteins in HUVECs. (**I**) Representative Western blot images showing the effects of FMO2 knockdown and overexpression on VCAM-1, ICAM-1, and NF-κB signaling in Ang II-stimulated HUVECs. (**J**) Quantitative analysis of VCAM-1, ICAM-1, and phosphorylated NF-κB relative to total NF-κB in the FMO2 gain- and loss-of-function experiments. (**K**) Representative Western blot images showing VCAM-1, ICAM-1, FMO2, and FTO expression in Ang II-stimulated HUVECs transfected with pcDNA, OE-FTO, or OE-FTO together with OE-FMO2. (**L**) Quantitative analysis of VCAM-1, ICAM-1, and FMO2 protein expression in the FTO overexpression experiment. All data are presented as mean ± SD (*n* = 3); * *p* < 0.05, ** *p* < 0.01, *** *p* < 0.001,**** *p* < 0.0001.

To investigate whether chemically characterized constituents of KJGLY contribute to its endothelial protective effects, we selected linarin, which showed the highest measured concentration among the quantified constituents, as a representative compound for preliminary cellular validation (Table S4). HUVECs were first exposed to increasing concentrations of Ang II to establish an endothelial injury model. Exposure to 200 nmol/L Ang II significantly reduced HUVEC viability and was therefore selected for subsequent experiments (Figure 7A). Linarin treatment significantly improved the viability of Ang II-injured HUVECs, with 50 μM producing the greatest protective effect (Figure 7B).

Based on these findings, Ang II-stimulated HUVECs were used to examine whether linarin modulates FMO2/FTO/m^6^A signaling. The transfection efficiencies of the FMO2 overexpression plasmid, FMO2 siRNA, and FTO overexpression plasmid were confirmed before subsequent experiments (Figure S5A–C). Ang II stimulation markedly reduced global m^6^A levels, whereas linarin partially restored m^6^A abundance. Concurrent FMO2 overexpression further enhanced this effect (Figure 7C). Western blot analysis showed that Ang II increased the expression of VCAM-1, ICAM-1, FTO, and phosphorylated NF-κB while decreasing FMO2 expression. Linarin treatment partially reversed these alterations, and FMO2 overexpression further attenuated the Ang II-induced inflammatory response (Figure 7D,F).

MeRIP-qPCR analysis revealed increased m^6^A enrichment of *VCAM-1* mRNA following FMO2 overexpression compared with the corresponding control group (Figure 7G). Co-immunoprecipitation analysis further demonstrated an association between endogenous FMO2 and FTO proteins in HUVECs (Figure 7H). To further evaluate the functional role of FMO2, gain- and loss-of-function experiments were performed. FMO2 silencing increased VCAM-1, ICAM-1, and phosphorylated NF-κB levels, whereas FMO2 overexpression produced the opposite effects (Figure 7I,J). Moreover, FTO overexpression increased VCAM-1 and ICAM-1 expression and reduced FMO2 abundance in Ang II-stimulated HUVECs. Concurrent overexpression of FMO2 partially counteracted these changes, as evidenced by reduced VCAM-1 and ICAM-1 levels and restored FMO2 expression compared with FTO overexpression alone (Figure 7K,L). Collectively, these findings indicate that linarin attenuates Ang II-induced endothelial inflammatory activation and support a functional association between FMO2, FTO, and m^6^A regulation.

## 4. Discussion

Hypertension is a major contributor to vascular remodeling and cardiovascular complications, largely through persistent endothelial dysfunction, inflammation, and oxidative stress. In the present study, KJGLY reduced blood pressure, improved vascular remodeling, attenuated inflammatory and oxidative responses, and restored endothelial homeostasis in SHRs. Our findings support FMO2 as a treatment-responsive protein associated with the vascular protective effects of KJGLY and suggest the potential involvement of FTO/m^6^A signaling.

Previous reports have identified the role of m^6^A RNA methylation in cardiovascular diseases [16,17]. FTO, an m^6^A demethylase, has recently attracted attention because of its reported association with hypertension [18,19,20]. However, no studies have yet delineated the upstream regulators of FTO in the context of hypertension. In this study, we conducted an unbiased proteomic analysis of SHRs and identified FMO2 as one of the most prominently altered proteins, implicating it in inflammatory and oxidative stress-related processes. FMO2, a member of the flavin-containing monooxygenase family, catalyzes oxidative reactions during xenobiotic metabolism [21], which helps reduce hepatic steatosis [22] and fibrosis [23]. FMO2 mediates cardiac fibrotic remodeling after myocardial infarction [24], lessens cardiomyocyte apoptosis [25], and diminishes immune cell infiltration-induced renal injury [26]. Because oxidative stress is a major contributor to hypertensive vascular injury [27], we further evaluated several indices of redox homeostasis. MDA is a reactive aldehyde generated during the peroxidation of polyunsaturated fatty acids and is widely used as an indicator of lipid peroxidation and membrane oxidative damage [28]. Excessive MDA accumulation may impair membrane integrity, promote protein modification, and aggravate endothelial dysfunction. In contrast, GSH is a major intracellular antioxidant, and glutathione peroxidase uses GSH as an electron donor to reduce hydrogen peroxide and lipid hydroperoxides, thereby limiting oxidative damage and maintaining cellular redox balance [29,30]. Consistent with these observations, FMO2 expression was reduced in hypertensive aortas and Ang II-stimulated endothelial cells, whereas restoration of FMO2 was associated with reduced inflammation and oxidative stress. KJGLY treatment decreased MDA levels while increasing GSH, SOD, and NO levels, suggesting attenuation of lipid peroxidation and restoration of endogenous antioxidant defenses. Conversely, AAV-mediated FMO2 knockdown aggravated vascular remodeling, endothelial activation, and oxidative injury in spontaneously hypertensive rats, supporting a protective role for FMO2 in vascular homeostasis during hypertension.

A major finding of the present study is the potential functional association between FMO2 and FTO/m^6^A regulation. KJGLY treatment increased FMO2 expression, reduced FTO abundance, and restored global m^6^A levels in hypertensive aortic tissues. In contrast, AAV9-mediated FMO2 knockdown was accompanied by increased FTO expression, reduced global m^6^A abundance, and enhanced inflammatory signaling. Furthermore, Co-IP analysis detected FMO2 and FTO within the same immunoprecipitated protein complex, supporting a protein-level association between them, whereas MeRIP-qPCR showed increased m^6^A enrichment of *VCAM-1* mRNA following FMO2 overexpression. In addition, FTO overexpression increased VCAM-1 and ICAM-1 expression in Ang II-stimulated endothelial cells, while concurrent FMO2 overexpression partially attenuated these changes. Together, these findings suggest that FMO2 may participate in the regulation of FTO-associated m^6^A modification, thereby influencing endothelial inflammatory activation. Given the established roles of VCAM-1 and ICAM-1 in endothelial activation, leukocyte adhesion, and vascular inflammation, suppression of these adhesion molecules is generally considered indicative of improved endothelial homeostasis. Their coordinated changes with FMO2, FTO, and global m^6^A levels further support a potential link between FMO2-associated m^6^A regulation and vascular inflammation in hypertension. Metabolomic profiling also revealed alterations in lipid-related pathways, particularly arachidonic acid metabolism, which has been closely associated with vascular inflammation. These findings provide an additional metabolic perspective on the response to KJGLY treatment; however, the functional contribution of individual lipid mediators was not directly examined in the present study. Based on the proteomic screening results and the convergent inflammatory and oxidative stress-related signatures observed across the omics datasets, FMO2 was prioritized for subsequent in vivo and in vitro functional investigation. Proteomic and transcriptomic analyses consistently identified enrichment of inflammatory and oxidative stress-related pathways, including NF-κB signaling. The convergence of these independent datasets suggests that KJGLY exerts coordinated regulatory effects on vascular inflammatory networks rather than acting through a single downstream target. Notably, FMO2 emerged as a common node linking these biological processes, supporting its role as a key regulator of vascular responses in hypertension.

The chemical characterization of KJGLY provides a material basis for its multi-target pharmacological activities. Among the quantified constituents, linarin showed the highest measured concentration and was therefore selected as a representative compound for further cellular validation. Previous studies have reported anti-inflammatory, antioxidant, and cardiovascular protective effects of linarin in various experimental models [31,32,33]. Consistent with these observations, linarin partially reproduced the effects of KJGLY in Ang II-injured endothelial cells, including restoration of FMO2/FTO signaling, increased m^6^A levels, and suppression of endothelial inflammatory activation. These findings suggest that linarin may contribute to the vascular protective effects of KJGLY. However, the synergistic contributions of other constituents cannot be excluded and warrant further investigation. Collectively, these observations provide additional support for the potential involvement of FMO2/FTO/m^6^A signaling in the vascular protective effects of KJGLY. Several limitations should be acknowledged. Although the additional FMO2 overexpression and FTO/FMO2 co-overexpression experiments in Ang II-stimulated HUVECs provide further functional evidence linking FMO2 to FTO/m^6^A-associated inflammatory regulation, the precise molecular mechanism by which FMO2 influences FTO expression or activity remains unresolved. The current data do not distinguish among direct protein association, transcriptional regulation, altered protein stability, or indirect metabolic effects. Moreover, the in vivo functional evidence was derived primarily from AAV9-mediated FMO2 knockdown, and an in vivo FMO2 overexpression or rescue experiment was not performed. Therefore, the reversibility of the vascular phenotype and the sufficiency of FMO2 restoration for vascular protection remain to be established. It is also unclear whether VCAM-1 is directly recognized or demethylated by FTO, and how FMO2-associated m^6^A alterations affect transcript stability, translation, and transcriptome-wide RNA methylation. AAV9-shFMO2 was administered systemically without a cell-type-specific promoter; accordingly, the relative contributions of endothelial cells, vascular smooth muscle cells, and other vascular populations remain unclear. The findings were obtained using a single SHR model and require validation in additional hypertensive models. Although linarin reproduced several protective effects in vitro, its contribution to the in vivo efficacy of the complete formula has not been established. Similarly, arachidonic acid-related metabolic alterations were identified by multi-omics analyses, but the functional roles of individual lipid mediators were not directly examined. Finally, the relevance of FMO2/FTO/m^6^A-related signaling to human hypertension remains to be determined.

## 5. Conclusions

In conclusion, KJGLY alleviated hypertensive vascular injury and attenuated vascular inflammation, oxidative stress, and remodeling in SHRs. Integrated multi-omics analyses together with in vivo and in vitro functional validation supported FMO2 as a treatment-responsive candidate protein associated with these effects. KJGLY treatment was accompanied by changes in FTO expression and global m^6^A levels, whereas linarin partially recapitulated the cellular effects of KJGLY in Ang II-stimulated endothelial cells. These findings provide new insight into the role of FMO2-dependent m^6^A regulation in hypertension-associated vascular injury and support further investigation of this pathway as a therapeutic target.

## Figures and Tables

**Table 1:** Detailed information about the formula of KJGLY.

Formula Components	*Ilex kudingcha*	*Chrysanthemum*	*Bidens pilosa*	*Pueraria lobata*	*Ligusticum chuanxiong*	*Stevia rebaudiana*
Traditional name in Chinese	Kudingcha	Yejuhua	Guizhencao	Gegen	Chuanxiong	Tianyeju
Scientific name of the component	*Ilex kaushue*	*Chrysanthemum indicum*	*Bidens pilosa*	*Pueraria montana* var. lobata	*Ligusticum chuanxiong*	*Stevia rebaudiana*
Family of the component	Aquifoliaceae	Asteraceae	Asteraceae	Fabaceae	Apiaceae	Asteraceae
Part used	Dried leaves	Dried flower	Dried whole plant	Dried roots	Dried rhizome	Dried leaves
Quantity in grams	30 g	30 g	30 g	15 g	15 g	8 g

## Data Availability

All the data in this paper are available from the corresponding author on request.

## Supplementary Materials

The following supporting information can be downloaded at: https://www.mdpi.com/article/10.3390/biomedicines14071469/s1, Figure S1. Total ion flow plots in buck-square; Figure S2. KEGG pathway enrichment and correlation analysis of differential metabolites; Figure S3. Functional classification of differentially expressed proteins and exploratory FMO2-centered metabolite correlation analysis; Figure S4. Hierarchical clustering heatmap of differential metabolites and protein–metabolite correlation analysis; Figure S5. Evaluation of FMO2 and FTO plasmid overexpression efficiency in HUVECs by Western blot analysis; Table S1. UPLC-Q-TOF/MS Analysis of KJGLY formula Chemical Composition; Table S2. The information of MRM for three analytes; Table S3. The primer sequences used for the study; Table S4. Chinese medicine simplex components of KJGLY were measured by HPLC-MS/MS.

## Abbreviations

The following abbreviations are used in this manuscript:

AAV	Adeno-Associated Virus
ACE	Angiotensin-Converting Enzyme
Ang II	Angiotensin II
DBP	Diastolic Blood Pressure
FMO2	Flavin-Containing Monooxygenase 2
FTO	Fat Mass and Obesity-Associated Protein
HUVECs	Human Umbilical Vein Endothelial Cells
HPLC–MS/MS	High-performance liquid chromatography–tandem mass spectrometry
ICAM-1	Intercellular Adhesion Molecule 1
IL-1β	Interleukin-1 Beta
IL-6	Interleukin-6
IL-10	Interleukin-10
KEGG	Kyoto Encyclopedia of Genes and Genomes
KJGLY	KuJiang GanLuoYin
Lin	Linarin
UPLC-Q-TOF/MS	Ultra-performance liquid chromatography–quadrupole time-of-flight mass spectrometry
MDA	Malondialdehyde
m^6^A	N6-Methyladenosine
MeRIP	Methylated RNA Immunoprecipitation
NF-κB	Nuclear Factor Kappa-Light-Chain-Enhancer of Activated B Cells
NO	Nitric Oxide
SBP	Systolic Blood Pressure
SHRs	Spontaneously Hypertensive Rats
SOD	Superoxide Dismutase
TCM	Traditional Chinese Medicine
VCAM-1	Vascular Cell Adhesion Molecule 1
WKY	Wistar–Kyoto Rats
