# Supplementary material for: KuJiang GanLuoYin Alleviates Hypertensive Vascular Injury and Modulates FMO2/FTO/m6A Signaling
Source: Biomedicines. 2026 Jun 28;14(7):1469. doi: 10.3390/biomedicines14071469 (PMC13403412; doi:10.3390/biomedicines14071469)
Supplement: Supplementary file 1 [file biomedicines-14-01469-s001.zip › Fig S1.pdf]

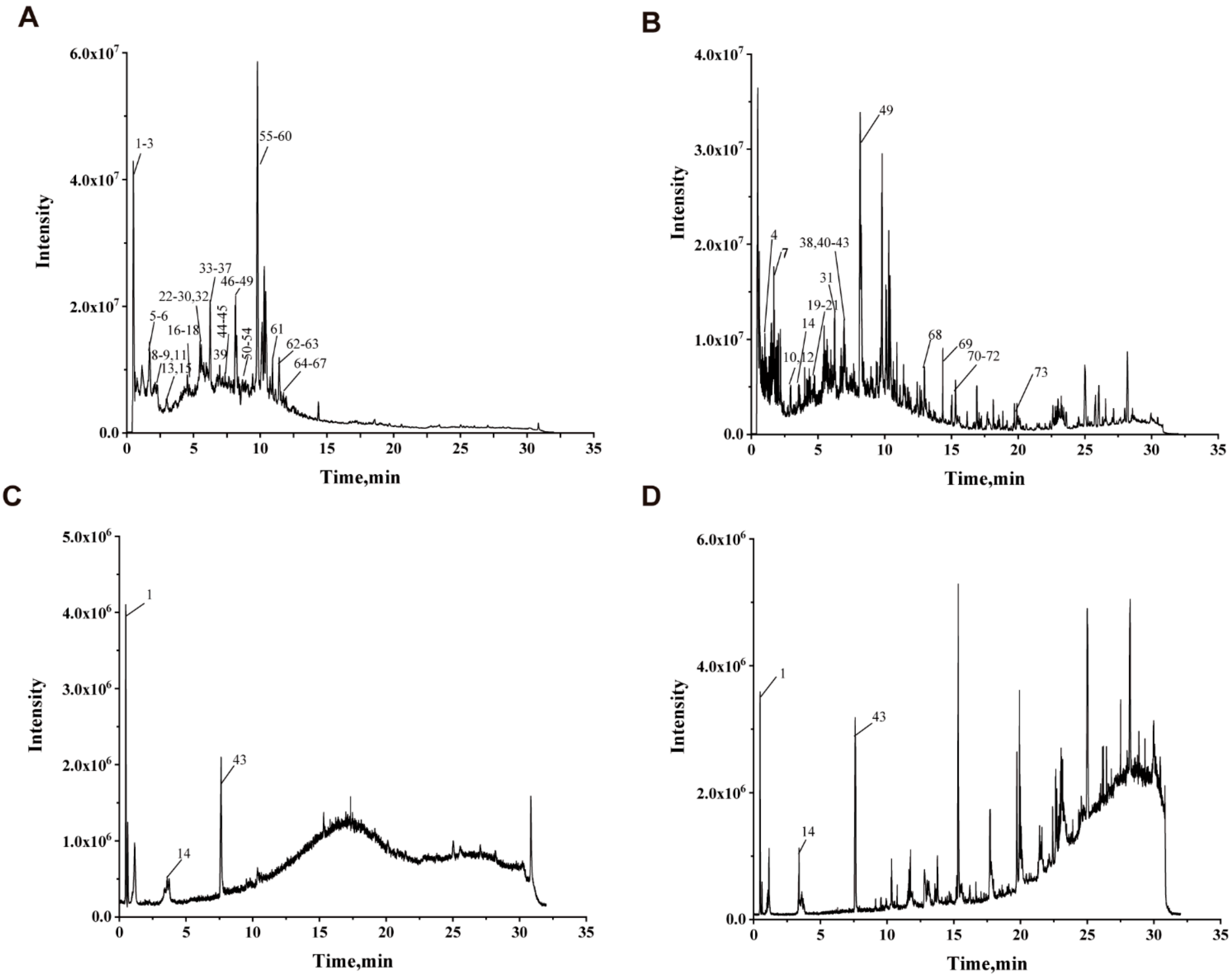

Figure S1 Total ion flow plots in buck-square.

(A) and negative (B) ion modes. Standard reference samples in positive (C) or negative (D) ion mode.

Peaks: 1-Gallic acid; 14-Ferulic acid; 43- Quercetin
