# Supplementary material for: KuJiang GanLuoYin Alleviates Hypertensive Vascular Injury and Modulates FMO2/FTO/m6A Signaling
Source: Biomedicines. 2026 Jun 28;14(7):1469. doi: 10.3390/biomedicines14071469 (PMC13403412; doi:10.3390/biomedicines14071469)
Supplement: Supplementary file 1 [file biomedicines-14-01469-s001.zip › Fig S2.pdf]

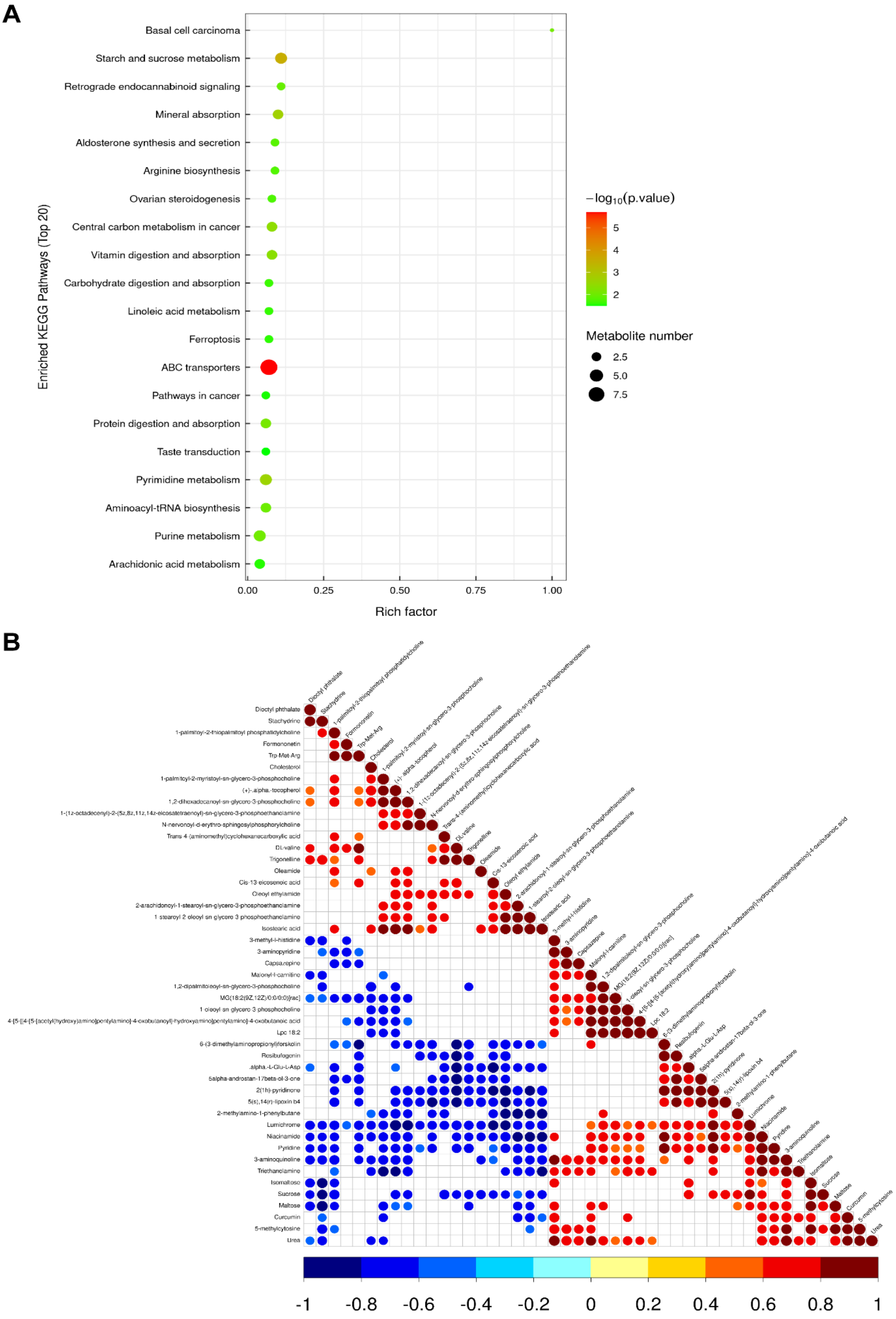

Figure S2 KEGG pathway enrichment and correlation analysis of differential

metabolites.

(A) KEGG enrichment analysis of significantly altered metabolic pathways. (B) Correlation matrix of differential metabolites; red indicates positive correlation, blue indicates negative correlation.
