# Supplementary material for: KuJiang GanLuoYin Alleviates Hypertensive Vascular Injury and Modulates FMO2/FTO/m6A Signaling
Source: Biomedicines. 2026 Jun 28;14(7):1469. doi: 10.3390/biomedicines14071469 (PMC13403412; doi:10.3390/biomedicines14071469)
Supplement: Supplementary file 1 [file biomedicines-14-01469-s001.zip › Fig S3.pdf]

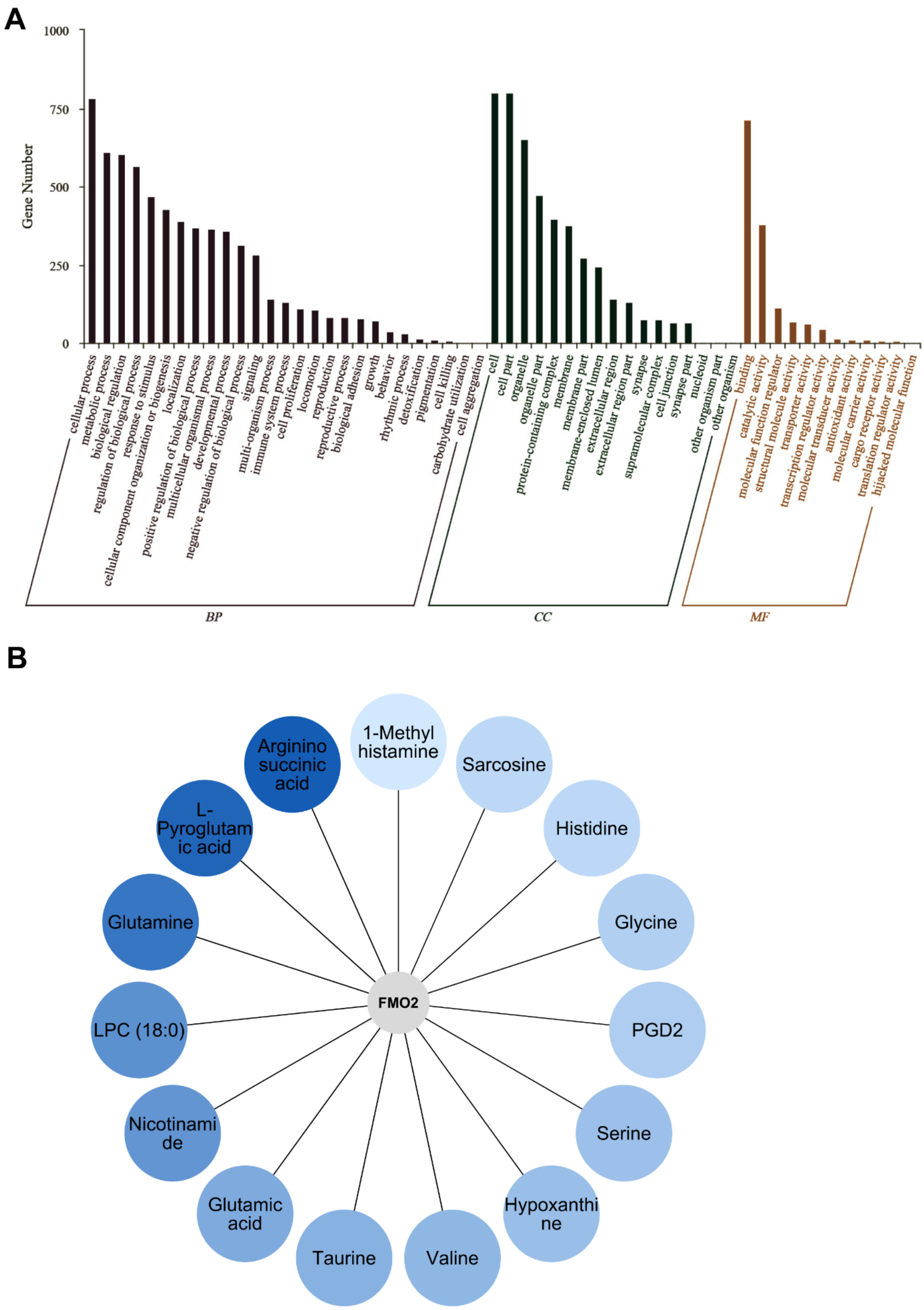

Figure S3 Functional classification of differentially expressed proteins and

exploratory FMO2-centered metabolite correlation analysis.

(A) GO pathways showing the top-enriched pathways identified in the proteomic analysis. (B) Exploratory FMO2-centered metabolite correlation network constructed by integrating proteomic and metabolomic data. Node color intensity represents the absolute correlation strength, with darker blue indicating a stronger correlation. Correlation analysis indicates statistical associations and does not establish a direct causal relationship.
