# Supplementary material for: KuJiang GanLuoYin Alleviates Hypertensive Vascular Injury and Modulates FMO2/FTO/m6A Signaling
Source: Biomedicines. 2026 Jun 28;14(7):1469. doi: 10.3390/biomedicines14071469 (PMC13403412; doi:10.3390/biomedicines14071469)
Supplement: Supplementary file 1 [file biomedicines-14-01469-s001.zip › Fig S4.pdf]

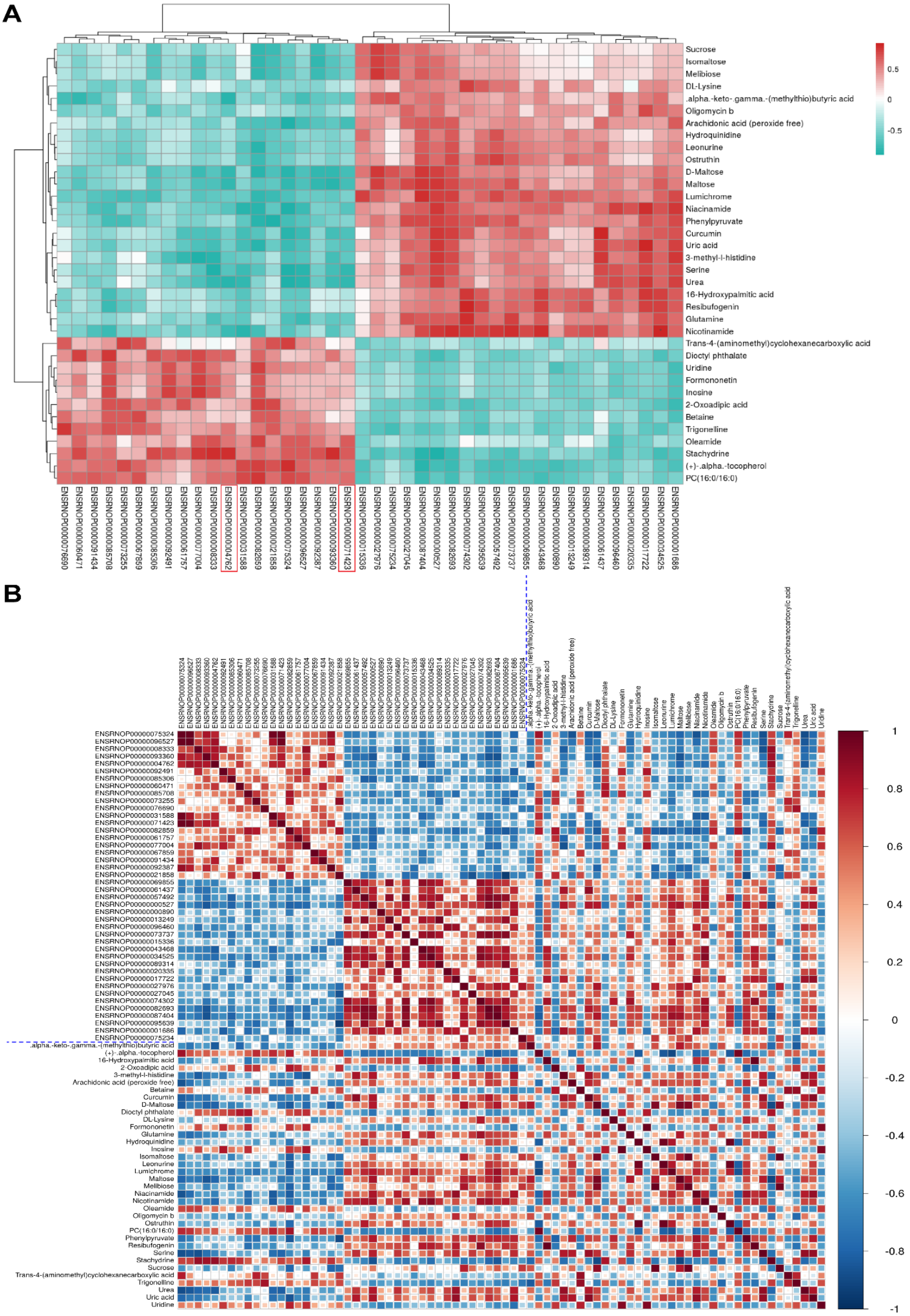

Figure S4 Hierarchical clustering heatmap of differential metabolites and protein–metabolite

correlation analysis.

(A) Correlation matrix of differential proteins and metabolites. (B) Clustered heatmap showing the integrated trends of metabolite–protein associations.
