# Supplementary material for: KuJiang GanLuoYin Alleviates Hypertensive Vascular Injury and Modulates FMO2/FTO/m6A Signaling
Source: Biomedicines. 2026 Jun 28;14(7):1469. doi: 10.3390/biomedicines14071469 (PMC13403412; doi:10.3390/biomedicines14071469)
Supplement: Supplementary file 1 [file biomedicines-14-01469-s001.zip › Fig S5.pdf]

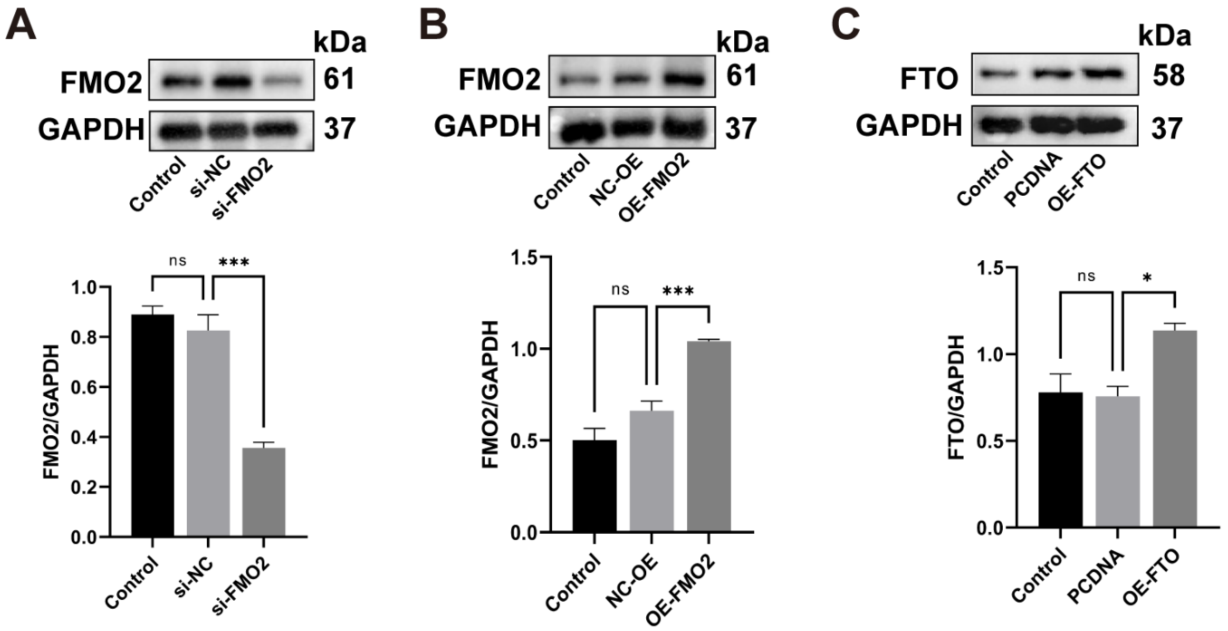

Figure S5 Evaluation of FMO2 and FTO plasmid overexpression efficiency in HUVECs by Western blot analysis.

(A) Representative western blot images showing transfection efficiency of FMO2 overexpression plasmid (n = 3). (B)Representative western blot images showing transfection efficiency of FMO2 knockdown plasmid (n = 3). (C)Representative Western blot images displaying transfection efficiency of FTO overexpression plasmid (n = 3). All data are presented as mean ± SD. *p < 0.05, ***p < 0.001; ns, no significant.
