# Supplementary material for: KuJiang GanLuoYin Alleviates Hypertensive Vascular Injury and Modulates FMO2/FTO/m6A Signaling
Source: Biomedicines. 2026 Jun 28;14(7):1469. doi: 10.3390/biomedicines14071469 (PMC13403412; doi:10.3390/biomedicines14071469)
Supplement: Supplementary file 1 [file biomedicines-14-01469-s001.zip › Supplemental Methods.pdf]

Supplemental Methods

2.1 KJGLY Preparation and UPLC-Q-TOF/MS Analysis

Gallic acid (Batch No.: 18032703) and chlorogenic acid (Batch No.: 21032502) were purchased from Chengdu Pufei De Biotech Co., Ltd. (Chengdu, China). Ferulic acid (Batch No.: RFS-A00211812016) was obtained from Chengdu Refine Biology Co., Ltd. (Chengdu, China), and quercetin (Batch No.: C12374611) was purchased from Shanghai Macklin Biochemical Co., Ltd. (Shanghai, China). All reference standards were of analytical grade for quantitative analysis, with a purity exceeding 98%. Appropriate amounts of each standard were accurately weighed and dissolved in methanol by ultrasonic treatment, and then mixed by ultrasonication before use. Methanol and acetonitrile (HPLC grade) were supplied by Thermo Fisher Scientific (USA). Formic acid was purchased from Merck (Germany). Ultrapure water was prepared using a Milli-Q system (Millipore, USA).

KJGLY comprises six natural medicinal components (Table 1). All herbal ingredients were provided by the Pharmaceutical Preparation Laboratory of Traditional Chinese Medicine at the Jiangsu Provincial Hospital of Traditional Chinese Medicine, and were authenticated by two senior herbalists. The components were weighed in a 30:30:30:15:15:8 ratio, soaked in 2 liters of double-distilled water for 30 minutes, and then boiled for an additional 30 minutes. This decoction was then mixed with an additional 2 liters of double-distilled water and boiled for an additional 30 minutes. The final decoction was then concentrated under reduced pressure (pressure: -0.04 to -0.01 MPa; temperature: 60 °C) until it had a relative density of 1.05. The supernatant was then centrifuged at 6,000 rpm for 15 minutes (flow rate: 4-6 L/min) to remove insoluble residues. The KJGLY extract was prepared according to traditional

decoction procedures and then concentrated using a rotary evaporator to a final density of 2.56 g/mL. An aliquot of 1 mL of the concentrated extract was accurately measured and mixed slowly with three volumes of absolute ethanol. The mixture was kept overnight at 4 °C to allow complete precipitation. The precipitate was removed by centrifugation at 12,000 rpm for 10 min. The supernatant was collected and evaporated to dryness using a vacuum concentrator. The resulting residue was reconstituted in 1 mL of 50% acetonitrile, vortexed for 5 min, and centrifuged again at 12,000 rpm for 10 min. The final supernatant was filtered through a 0.22 µm membrane filter before injection.

Chromatographic separation was performed on an ACQUITY UPLC BEH C18 column (2.1 mm × 100 mm, 1.7 µm; Waters, USA) maintained at 40 °C. The mobile phase consisted of solvent A (0.1% formic acid in water, v/v) and solvent B (acetonitrile). The flow rate was set at 0.4 mL/min and the injection volume was 4 µL. The gradient elution program was as follows: 0–2 min, 10% B; 2–28 min, linear increase from 10% to 80% B; 28–30 min, 80% B; 30–30.1 min, decrease from 80% to 10% B; 30.1–32 min, 10% B. The run was stopped at 32 min.

Mass spectrometric detection was conducted using an electrospray ionization (ESI) source operated in both positive and negative ionization modes. The source parameters were set as follows: ion source temperature (TEM), 550 °C; curtain gas (CUR), 35 psi; nebulizer gas (GS1), 55 psi; auxiliary gas (GS2), 55 psi; ion spray voltage (IS), +5500 V/−5500 V; collision energy (CE), ±10 V; declustering potential (DP), ±80 V. The mass scan range was set at m/z 100–1250.

2.2 Quantitative HPLC-MS/MS Analysis of Representative Constituents in KJGLY

Quantitative HPLC-MS/MS Analysis of Representative Constituents in KJGLY

Quantitative analysis of selected constituents in KJGLY was performed using an ExionLC™ AC high-performance liquid chromatography system coupled to a ZenoTOF™ 7600 quadrupole time-of-flight mass spectrometer (AB SCIEX, Framingham, MA, USA). Chromatographic separation was achieved on an ACQUITY UPLC BEH C18 column (2.1 mm × 100 mm, 1.7 μm; Waters, Milford, MA, USA) maintained at 40 °C. The mobile phases consisted of water containing 0.1% formic acid (A) and acetonitrile (B). The flow rate was 0.4 mL/min, and the injection volume was 4 μL. The gradient elution program was as follows: 0–2 min, 10% B; 2–28 min, 10–80% B; 28–30 min, 80% B; 30–30.1 min, 80–10% B; and 30.1–32 min, 10% B.

Mass spectrometric detection was conducted using an electrospray ionization source in both positive and negative ion modes. The source temperature was set at 550 °C, with curtain gas at 35 psi, nebulizer gas at 55 psi, and auxiliary heating gas at 55 psi. The ion spray voltages were set at +5500 V and −5500 V for positive- and negative-ion acquisition, respectively. Survey mass spectra were acquired over an m/z range of 100–1250. Targeted MS/MS quantification was performed using compound-specific precursor-to-product ion transitions. The optimized declustering potential and collision energy values for each analyte are provided in Table S4.

The same concentrated KJGLY aqueous decoction used for UPLC-Q-TOF/MS characterization was used for quantitative analysis. Briefly, 1 mL of the concentrated decoction was mixed slowly with three volumes of absolute ethanol and maintained at 4 °C overnight to precipitate macromolecular components. The mixture was centrifuged at 12,000 rpm for 10 min at 4 °C, and the resulting supernatant was collected and evaporated to dryness using a SpeedVac centrifugal concentrator (Thermo Scientific, Waltham, MA, USA). The residue was reconstituted in 1 mL of

50% acetonitrile, vortexed for 5 min, and centrifuged again at 12,000 rpm for 10 min at 4 °C. The supernatant was transferred to an autosampler vial for analysis.

Reference standards (Shanghai, China) of gallic acid, hyperoside, rutin, ferulic acid, calycosin, luteolin, naringenin, quercetin, kaempferol, ligustrazine phosphate, senkyunolide A, apigenin, and linarin were used for quantitative analysis. All reference compounds had a stated purity of at least 98%.

Methanol and acetonitrile of chromatographic grade were obtained from Merck (Darmstadt, Germany). Formic acid of chromatographic grade was purchased from Aladdin Reagent Co., Ltd. (Shanghai, China), and analytical-grade absolute ethanol was obtained from Nanjing Chemical Reagent Co., Ltd. (Nanjing, China). Ultrapure water was generated using a Milli-Q purification system (Millipore, USA).

Individual standard stock solutions were prepared in methanol and mixed in appropriate proportions. The mixed standard solution was serially diluted with methanol to prepare calibration solutions at different concentration levels. The concentrations of the analytes in the KJGLY test solution were calculated using the respective external-standard calibration curves based on chromatographic peak areas. The precursor/product ion transitions, declustering potentials, collision energies, and calculated concentrations are summarized in Table A4.

2.3 Animals

All experimental protocols were approved by the Animal Ethics Committee of the Jiangsu Provincial Hospital of Traditional Chinese Medicine (approval no. 2024DW-023-01, 11 March 2024). To eliminate the potential confounding effects of oestrogen on cardiovascular outcomes, this study only included 9-week-old male SHRs and Wistar-Kyoto (WKY) rats. All rats (200–220 g) were obtained from Beijing Vital River Laboratory Animal Technology Co., Ltd. (Licence No. SCXK (Beijing) 2021-

0006) and were housed in a temperature-controlled room (25 ± 1 °C) with free access to water and standard feed.

For the pharmacodynamic experiment, the WKY rats served as the control group and were administered distilled water via oral gavage. The SHRs were randomly divided into four groups and administered equal volumes of distilled water, valsartan (8.5 mg/kg/day), or KJGLY (6.82 g/kg/day or 13.65 g/kg/day) via oral gavage once daily for eight consecutive weeks. These doses were converted based on the body surface area ratio between rats and humans, whereby the daily dose for rats equalled 6.4 times that for a human adult (60 kg). Specifically, the clinical doses of valsartan and KJGLY were 8.5 mg/kg/day and 1.4 g/kg/day, respectively (calculated as 80 mg/60 kg/day*6.4 = 8.5 mg/kg/day and 128 mg/60 kg/day*6.4 = 13.65 mg/kg/day). Systolic blood pressure was measured using an intelligent, non-invasive blood pressure monitor (Softron, Japan) at baseline and at 2-, 4-, 6- and 8-week post-treatment. The rats were then anaesthetised with 0.3% sodium pentobarbital (0.1 mL/100 g, intraperitoneally). Blood samples were collected from the abdominal aorta, after which the rats were euthanised. Aortic tissues were harvested and subjected to histological analysis according to standardised protocols.

For the mechanistic study, a recombinant adeno-associated virus (AAV) with a viral titer of 1 × 10^{13} v.g./mL was synthesised. Nine-week-old SHRs were randomly divided into two groups and inoculated via tail vein injection at a dose of 0.1 mL per 100 g of body weight. The first group received a single injection of AAV9-NC + normal saline, and the second group received a single injection of AAV9-FMO2-shRNA. As peak in vivo expression was expected three weeks after injection, the rats were anaesthetised with 0.3% pentobarbital sodium (0.1 mL/100 g, intraperitoneal injection) at this time point, after which blood samples were collected from the

abdominal aorta before euthanasia. The aortas were then harvested and subjected to histological analysis following standard procedures.

2.4 Cell culture and Plasmid Transfection

Human umbilical vein endothelial cells (HUVECs; RRID: CVCL_2959) were purchased from Procell Life Science & Technology Co., Ltd. (Wuhan, China) and routinely verified to be free of mycoplasma contamination. Cells were maintained in complete DMEM supplemented with fetal bovine serum, antibiotics, and endothelial cell growth factor under standard culture conditions (37 °C, 5% CO_2).

For gain- and loss-of-function experiments, HUVECs were transfected with FMO2 overexpression plasmids (OE-FMO2), FMO2-specific siRNA (si-FMO2), FTO overexpression plasmids (OE-FTO), or corresponding control vectors obtained from OBIO (Shanghai, China). Transfections were performed using Lipofectamine 3000 (Invitrogen) according to the manufacturer's instructions.

2.15 Histopathological Analyses

Aortic tissues were collected from rats and fixed in 4% paraformaldehyde for subsequent histological assessment. The samples were sectioned at a thickness of 4 μm and stained with hematoxylin–eosin (H&E) and Masson's trichrome to evaluate tissue morphology and fibrosis. Aortic wall thickness was quantified using CaseViewer (v2.4.0.119028), and fibrotic areas were calculated relative to the normal regions using ImageJ software.

For immunohistochemistry (IHC), the tissue sections were dehydrated, subjected to heat-induced antigen retrieval in citric acid buffer, and incubated with primary antibodies targeting vascular cell adhesion molecule 1 (VCAM-1) (66294-1-Ig, Proteintech, 1:200) and intercellular adhesion molecule 1(ICAM-1) (10020-1-AP, Proteintech, 1:200). The samples were then visualized and scanned.

Immunofluorescence (IF) analysis involved overnight incubation at 4 °C with anti-FMO2 (DF16081, Affbiotech, 1:100) and anti-FTO (27226-1-AP, Proteintech, 1:200), followed by HRP-conjugated secondary antibody incubation at 37 °C for 1 h. The sections were washed with PBS and counterstained with DAPI for nuclear visualization.
