# Supplementary material for: KuJiang GanLuoYin Alleviates Hypertensive Vascular Injury and Modulates FMO2/FTO/m6A Signaling
Source: Biomedicines. 2026 Jun 28;14(7):1469. doi: 10.3390/biomedicines14071469 (PMC13403412; doi:10.3390/biomedicines14071469)
Supplement: Supplementary file 1 [file biomedicines-14-01469-s001.zip › Table S1.pdf]

Table S1. UPLC-Q-TOF/MS Analysis of KJGLY formula Chemical Composition

No.	RT (min)	Molecular Formula	Compound	Theoretical Value	Measured Value	Secondary Fragment Ions	Error (ppm)	Ion Mode	Source
**1**	0.63	C7H6O5	Neochlorogenic acid	169.0143	169.0139		-2.3	[M-H]-	Bidens pilosa kudingcha
**2**	0.80	C16H18O9	Neochlorogenic acid	353.0878	353.0878	191.0572, 179.0357, 135.0460	-2.6	[M-H]-	Chrysanthemum, Ilex kudingcha
**3**	0.84	C7H6O4	Protocatechuic acid	153.0193	153.0191	109.0349, 91.0194	-1.8	[M-H]-	Ilex kudingcha, Bidens pilosa
**4**	1.14	C16H18O9	Chlorogenic acid	355.1024	355.1024	163.0473, 145.0333, 135.0470, 117.0358	0.1	[M+H]+	Chrysanthemum, Ilex kudingcha
**5**	1.38	C9H8O4	Caffeic acid	179.0350	179.0523	135.0553, 117.0350, 89.0401	-2.2	[M-H]-	Ligusticum chuanxiong, Pueraria lobata
**6**	1.64	C8H8O4	Vanillic acid	167.0350	167.0343	121.0297	-3.9	[M-H]-	Ligusticum chuanxiong
**7**	1.66	C21H20O9	Puerarin	417.1180	417.1182	399.1112, 381.1004, 363.0388, 351.0895, 321.0788, 297.0835, 267.0723	0.5	[M+H]+	Pueraria lobata
**8**	2.07	C22H22O10	Methoxypuerarin	445.1140	445.1137	325.0732, 297.0769	-0.6	[M-H]-	Pueraria lobata
**9**	2.28	C26H28O13	Puerarin xyloside	547.1457	547.1452	295.0623, 267.0670	-1.0	[M-H]-	Pueraria lobata

**10**	2.57	C9H6O2	1,2-Indanedione	147.0441	147.0440	147.0449, 119.0486, 91.0538	-0.5	[M+H]+	Pueraria lobata
**11**	2.63	C9H8O3	3-Hydroxycinnamic acid	163.0401	163.0397	119.0562, 93.0350	-2.5	[M-H]-	Pueraria lobata
**12**	2.95	C21H20O9	Daidzin	417.1180	417.1187	255.0734	1.7	[M+H]+	Pueraria lobata
**13**	3.15	C21H20O10	3'-Hydroxypuerarin	431.0984	431.0983	311.0560, 283.0627	-2.5	[M-H]-	Pueraria lobata
**14**	3.53	C10H10O4	Ferulic acid	195.0652	195.0650	177.0549, 145.0300, 117.0337, 89.0404	-1.2	[M+H]+	Ligusticum chuanxiong
**15**	3.70	C16H12O5	Maackiain	283.0612	283.0606	239.0350, 211.0398, 91.0184	-2.2	[M-H]-	Pueraria lobata
**16**	4.04	C26H28O14	Isoxanthohumol	563.1406	563.1403	341.0692, 311.0699, 283.0702	-0.6	[M-H]-	Pueraria lobata
**17**	4.47	C21H20O12	Hyperoside	463.0882	463.0877	301.0358, 287.0642, 271.0248	-1.1	[M-H]-	Bidens pilosa
**18**	4.54	C27H30O16	Rutin	609.1461	609.1459	301.03671	-0.3	[M-H]-	Bidens pilosa
**19**	4.73	C21H20O10	Dye wood glycoside	433.1129	433.1132	271.0698	0.7	[M+H]+	Pueraria lobata
**20**	4.95	C12H18O4	Ligustilide N	227.1278	227.1275	209.1164, 191.1063, 163.1110, 91.0536	-1.4	[M+H]+	Ligusticum chuanxiong
**21**	4.96	C12H16O3	Ligustilide G	209.1172	209.1170	191.1060, 177.0535, 163.1109, 145.0998	-1	[M+H]+	Ligusticum chuanxiong
**22**	5.40	C30H36O15	Puerarin B	681.2025	681.2020	473.1519, 311.0933, 267.1053	-0.8	[M+HCOO]-	Pueraria lobata
**23**	5.47	C25H24O12	Dihydrochlorogenic acid B	515.1195	515.1188	353.0896, 335.0776, 191.0566, 173.0466	-1.4	[M-H]-	Chrysanthemum, Ilex kudingcha

**24**	5.00	C27H30O15	Luteolin 7-O-glucoside	593.1512	593.1499	285.0417, 255.0303	-2.5	[M-H]-	Chrysanthemum
**25**	5.53	C27H30O15	Nicotiflorosid e	593.1512	593.1512	285.0450, 255.0295	-2.2	[M-H]-	Pueraria lobata
**26**	5.58	C16H18O9	Cryptochlorogenic acid	353.0878	353.0868	191.0616, 173.0460, 135.0479	-2.8	[M-H]-	Chrysanthemum
**27**	5.67	C9H14O	2-Pentylfuran	137.0972	137.0967	121.0659, 93.0343	-3.3	[M-H]-	Ligusticum chuanxiong
**28**	5.79	C28H32O16	Narcissin	623.1618	623.1609	577.1622, 315.0646, 269.0558	-1.3	[M-H]-	Pueraria lobata
**29**	5.79	C27H30O14	Apigenin 7-O-glucoside	577.1563	577.1550	269.0575	-2.3	[M-H]-	Chrysanthemum
**30**	5.83	C21H30O11	Ilexperphenoside A	503.1759	503.1756	293.0885, 191.0561, 149.0459, 89.0249	-0.7	[M+HCOO]-	Ilex kudingcha
**31**	5.93	C21H20O10	Chrysoeriol 7-O-glucoside	433.1129	433.1131	271.0704	0.4	[M+H]+	Chrysanthemum
**32**	5.94	C21H18O11	Apigenin 7-O-glucuronide	445.0776	445.0764	269.0481, 175.0247	-2.8	[M-H]-	Chrysanthemum
**33**	6.24	C9H6O3	Umbelliferon e	161.0244	161.0238	133.0322	-3.7	[M-H]-	Pueraria lobata
**34**	6.26	C25H24O12	Dihydrochlorogenic acid A	515.1195	515.1189	353.0912, 191.0571, 173.0475	-0.6	[M-H]-	Chrysanthemum, Ilex kudingcha
**35**	6.30	C28H32O15	Diosmin	607.1668	607.1651	299.0673, 284.0349	-2.8	[M-H]-	Pueraria lobata
**36**	6.34	C24H22O14	Luteolin 7-O-propionylglucoside	533.0937	533.0923	327.0505, 285.0487	-2.6	[M-H]-	Chrysanthemum
**37**	6.45	C21H20O11	Luteoloside	447.0933	447.0918	285.0418	-3.4	[M-H]-	Bidens pilosa
**38**	6.79	C24H26O10	Puerarin C	475.1599	475.1603	295.0976, 267.1029, 253.0865	0.9	[M+H]+	Pueraria lobata

**39**	6.92	C15H10O4	3,3'-Dihydroxyflavone	253.0506	253.0504	225.0568, 133.0302	-0.8	[M-H]-	Pueraria lobata
**40**	6.96	C22H22O9	Mangiferin	431.1337	431.1340	269.0893	0.7	[M+H]+	Pueraria lobata
**41**	7.40	C29H34O15	Salidroside	623.1971	623.1976	477.1397, 315.0864	0.9	[M+H]+	Pueraria lobata
**42**	7.42	C16H12O5	Acacetin	285.0758	285.0759	253.0496, 225.0542	0.4	[M+H]+	Chrysanthemum
**43**	7.60	C15H10O7	Quercetin	303.0499	303.0502	229.0489, 153.0175, 137.0233	1.0	[M+H]+	Bidens pilosa
**44**	7.66	C15H10O6	Luteolin	285.0405	285.0402	217.0525, 199.0422, 175.0434, 151.0082, 133.0405	-0.9	[M-H]-	Chrysanthemum, Bidens pilosa
**45**	7.68	C10H18O4	Decanedioic acid	201.1132	201.1126	183.1033, 139.1143	-3.2	[M-H]-	Pueraria lobata
**46**	8.14	C28H32O14	Mongoloside	591.1719	591.1704	283.0711, 268.0389	-2.5	[M-H]-	Chrysanthemum
**47**	8.40	C16H12O4	Puncturevine steroidal glycoside	267.0663	267.0657	251.0360, 223.0447, 195.0478	-2.4	[M-H]-	Pueraria lobata
**48**	8.61	C16H12O5	3'-Methoxy-genistein	283.0612	283.0602	267.0311, 211.0426	-3.6	[M-H]-	Pueraria lobata
**49**	8.71	C12H8O4	5-Methoxy-psoralen	217.0495	217.0497	174.0305	0.5	[M+H]+	Ligusticum chuanxiong
**50**	8.94	C15H10O5	Apigenin	269.0456	269.0449	225.0563, 149.0255, 117.0420	-2.3	[M-H]-	Chrysanthemum
**51**	8.99	C53H84O23	Kudinoside C	1133.5375	1133.5349	1087.5323, 925.4776, 729.6907	-2.3	[M+HCOO]-	Ilex kudingcha
**52**	9.35	C16H12O6	Genkwanin	299.0561	299.0551	284.0377, 256.0386	-3.2	[M-H]-	Pueraria lobata
**53**	9.44	C11H20O4	Undecanedioic acid	215.1289	215.1281	197.1201, 153.1291	-3.7	[M-H]-	Pueraria lobata
**54**	9.78	C44H70O23	Rebudioside A	965.4235	965.4221	965.4237, 803.3716	-1.5	[M-H]-	Stevia rebaudiana

**55**	10.11	$C_{47}H_{76}O_{17}$	Ilexoside A	957.5054	957.5045	911.5110, 749.4507	-0.8	[M+HCOO]-	Ilex kudingcha
**56**	10.12	$C_{53}H_{86}O_{22}$	Kudinoside G	1119.5582	1119.5570	1073.5545, 911.5088	-1.1	[M+HCOO]-	Ilex kudingcha
**57**	10.23	$C_{41}H_{66}O_{13}$	Kudinoside H	765.4431	765.4408	719.4415, 603.3905, 509.4175	-3.0	[M-H]-	Ilex kudingcha
**58**	10.30	$C_{44}H_{70}O_{22}$	Rebudioside C	949.4286	949.4274	787.3849	-1.3	[M-H]-	Stevia rebaudiana
**59**	10.33	$C_{32}H_{50}O_{13}$	Stevioside	641.3179	641.3163	479.2643	-2.4	[M-H]-	Stevia rebaudiana
**60**	10.50	$C_{38}H_{60}O_{18}$	Steviolbioside	803.3707	803.3691	641.3296	-2.0	[M-H]-	Stevia rebaudiana
**61**	11.00	$C_{32}H_{50}O_{14}$	Dulcoside A	641.3179	641.3162	479.2656	-2.5	[M-H]-	Stevia rebaudiana
**62**	11.71	$C_{47}H_{72}O_{17}$	Kudinoside D	907.4697	907.4666	745.4620, 585.6025, 415.1359	-3.4	[M-H]-	Ilex kudingcha
**63**	11.89	$C_{12}H_{12}O_3$	Ligustilide B	203.0714	203.0704	173.0244, 145.0295	-4.8	[M-H]-	Ligusticum chuanxiong
**64**	12.20	$C_{47}H_{74}O_{18}$	Kudinoside A	925.4802	925.4777	616.1796, 601.5243	-2.7	[M-H]-	Ilex kudingcha
**65**	12.25	$C_{53}H_{86}O_{21}$	Ilekudinoside A	1103.5633	1103.5608	1057.5547, 895.5084, 735.1770	-2.2	[M+HCOO]-	Ilex kudingcha
**66**	12.46	$C_{12}H_{18}O_3$	Sedanonic acid	209.1183	209.1174	165.5550, 121.0301	-4.2	[M-H]-	Ligusticum chuanxiong
**67**	12.51	$C_{48}H_{78}O_{18}$	Apiosylskimmin J	941.5115	941.5084	895.5068, 733.4537	-3.3	[M-H]-	Ilex kudingcha
**68**	12.97	$C_{12}H_{16}O_2$	Ligustilide A	193.1223	193.1222	175.1141, 147.1221, 137.0672, 105.0719, 91.0611	-0.4	[M+H]+	Ligusticum chuanxiong

**69**	14.36	C32H40O8	Chrysanthemolide	553.2796	553.2793	493.2575, 475.2499, 451.2476, 229.1209, 151.1007	-0.5	[M+H]+	Chrysanthemum
**70**	14.89	C12H18O2	New Ligustilide	195.1380	195.1374	177.1273, 149.1313, 125.0591, 107.0849, 93.0691	-3.0	[M+H]+	Ligusticum chuanxiong
**71**	14.98	C12H14O2	Cnidilide	191.1067	191.1065	173.0985，145.1003，117.0704，91.0549	-0.7	[M+H]+	Ligusticum chuanxiong
**72**	15.00	C12H14O2	Butylphthalide	191.1067	191.1065	173.0980, 145.1010, 117.0696, 91.0553	-0.8	[M+H]+	Ligusticum chuanxiong
**73**	20.39	C24H28O4	Angelol A	381.2060	381.2059	191.1075, 173.0953, 135.0435, 91.0535	-0.5	[M+H]+	Ligusticum chuanxiong
