# Supplementary material for: KuJiang GanLuoYin Alleviates Hypertensive Vascular Injury and Modulates FMO2/FTO/m6A Signaling
Source: Biomedicines. 2026 Jun 28;14(7):1469. doi: 10.3390/biomedicines14071469 (PMC13403412; doi:10.3390/biomedicines14071469)
Supplement: Supplementary file 1 [file biomedicines-14-01469-s001.zip › Table S2.pdf]

Table S2. The information of MRM for three analytes.

Compound	Formula	RT(min)	Identification	Theoretical	Measured	MS/MS fragments	Error(ppm)	Ion mode
Gallic acid	C7H6O5	0.62	170.02152	171.0288	171.02852	153.0189<br>125.0227<br>107.0128<br>97.0283<br>81.0339	-1.6	[M+H]+
Ferulic acid	C10H10O4	3.34	194.05791	195.06519	195.0655	145.0282<br>117.0332<br>89.0384	-1.6	[M+H]+
Quercetin	C15H10O7	7.61	302.04265	303.05002	303.04993	285.0394<br>229.0506<br>153.0191	0.3	[M+H]+
