# Supplementary material for: KuJiang GanLuoYin Alleviates Hypertensive Vascular Injury and Modulates FMO2/FTO/m6A Signaling
Source: Biomedicines. 2026 Jun 28;14(7):1469. doi: 10.3390/biomedicines14071469 (PMC13403412; doi:10.3390/biomedicines14071469)
Supplement: Supplementary file 1 [file biomedicines-14-01469-s001.zip › Table S3.pdf]

Table S3. The primer sequences used for the study.

Primer name	5’>3’
Merip-VCAM-1 (human)	Forward: AAGCTGTGAGCTGGGAAGAG
	Reverse: CTCTCCCAGGACTCCAGGT
IL-6 (Rat)	Forward:GAGTTGTGCAATGGCAATTCTG
	Reverse: ACGGAACTCCAGAAGACCAGAG
IL-10 (Rat)	Forward:CAGACCCACATGCTCCGAGA
	Reverse: TGGCAACCCAAGTAACCCTTA
IL-1β (Rat)	Forward:GCTCGCCAGTGAAATGATGG
	Reverse: CTGGAAGGAGCACTTCATCTGT
GAPDH (Rat)	Forward:CTGGAGAAACCTGCCAAGTATG
	Reverse: GGTGGAAGAATGGGAGTTGCT
