# Supplementary material for: KuJiang GanLuoYin Alleviates Hypertensive Vascular Injury and Modulates FMO2/FTO/m6A Signaling
Source: Biomedicines. 2026 Jun 28;14(7):1469. doi: 10.3390/biomedicines14071469 (PMC13403412; doi:10.3390/biomedicines14071469)
Supplement: Supplementary file 1 [file biomedicines-14-01469-s001.zip › Table S4.pdf]

Table S4. Chinese medicine simplex components of KJGLY were measured by HPLC-MS/MS.

Standard	m/z	DP (V)	CE (V)	Concentration (ng/mL)
Gallic acid	168.8 → 125.1	70	27.86	22400
Hyperoside	463.1 → 300.1	80	40	24500
Rutin	609.1 → 300	110	50	186000
Ferulic acid	193.2 → 134.1	67	18	56000
Calycosin	283.1 → 268.1	21	24	443
Luteolin	285 → 133	80	34	3300
Naringenin	271 → 119.1	120	34	1010
Quercetin	300.8 → 150.8	138.94	27.05	6650
Kaempferol	284.8 → 185.1	148	34.98	275
Ligustrazine phosphate	134 → 116	94.85	11.71	161
Senkyunolide A	193.3 → 134.1	59	20.26	479
Apigenin	271.3 → 153.3	111	51	3530
Linarin	593.55 → 447.5	80	30	1220000
